# Association between Striatal Brain Iron Deposition, Microbleeds and Cognition 1 Year After a Minor Ischaemic Stroke

**DOI:** 10.3390/ijms20061293

**Published:** 2019-03-14

**Authors:** Maria del C. Valdés Hernández, Tessa Case, Francesca M. Chappell, Andreas Glatz, Stephen Makin, Fergus Doubal, Joanna M. Wardlaw

**Affiliations:** 1 College of Medicine and Veterinary Medicine, University of Edinburgh, Edinburgh EH16 4SB, UK; F.Chappell@ed.ac.uk (F.M.C.); andi.glatz@gmail.com (A.G.); Stephen.Makin@glasgow.ac.uk (S.M.); fergus.doubal@ed.ac.uk (F.D.); Joanna.Wardlaw@ed.ac.uk (J.M.W.); 2 Department of Neuroimaging Sciences, Centre for Clinical Brain Sciences, University of Edinburgh, Edinburgh EH16 4SB, UK; 3 Dementia Research Institute, University of Edinburgh, Edinburgh EH16 4SB, UK; 4 Row Fogo Centre for Ageing and the Brain, University of Edinburgh, Edinburgh EH16 4SB, UK; tessacase@hotmail.com

**Keywords:** iron deposits, MRI, ageing, cognition, brain microbleeds, white matter hyperintensities

## Abstract

Brain iron deposits (IDs) are inversely associated with cognitive function in community-dwelling older people, but their association with cognition after ischemic stroke, and whether that differs from microbleeds, is unknown. We quantified basal ganglia IDs (BGID) and microbleeds (BMBs) semi-automatically on brain magnetic resonance images from patients with minor stroke (NIHSS < 7), at presentation and 12 months after stroke. We administered the National Adult Reading Test (NART, estimates premorbid or peak adult cognition) and the Revised Addenbrooke’s Cognitive Examination (ACE-R; current cognition) at 1 and 12 months after stroke. We adjusted analyses for baseline cognition, age, gender, white matter hyperintensity (WMH) volume and vascular risk factors. In 200 patients, mean age 65 years, striatal IDs and BMBs volumes did not change over the 12 months. Baseline BGID volumes correlated positively with NART scores at both times (ρ = 0.19, *p* < 0.01). Baseline and follow-up BGID volumes correlated positively with age (ρ = 0.248, *p* < 0.001 and ρ = 0.271, *p* < 0.001 respectively), but only baseline (and not follow-up) BMB volume correlated with age (ρ = 0.129, *p* < 0.05). Both smoking and baseline WMH burden predicted verbal fluency and visuospatial abilities scores (B = −1.13, *p* < 0.02 and B = −0.22, *p* = 0.001 respectively) at 12 months after stroke. BGIDs and BMBs are associated differently with cognition post-stroke; studies of imaging and post-stroke cognition should adjust for premorbid cognition. The positive correlation of BGID with NART may reflect the lower premorbid cognition in patients with stroke at younger vs older ages.

## 1. Introduction

Brain mineral deposits are visible on susceptibility-weighted MRI and are associated with cognitive decline in ageing [1,2]. In community dwelling subjects, striatal iron deposition (ID) explains up to 9% of the variance in cognitive ability in old age and is also related to cognitive ability [3]. In-vivo and ex-vivo studies demonstrate that this iron is located in the walls of the perforating arterioles in the striatum [4,5], and the association between striatal iron deposits and cognition suggests a possible relationship between mineral deposits and microvascular disease. Brain iron accumulation has also been associated with high cholesterol intake and high plasma cholesterol, suggesting that high blood cholesterol may reduce the integrity of the blood-brain-barrier and/or disrupts iron metabolism in ways that render the brain vulnerable to cholesterol-related cellular stress [6]. However, there are no studies of the impact of IDs on cognition after stroke, although poor outcome after stroke has been related to high levels of iron in plasma and cerebrospinal fluid [7,8].

Brain microbleeds (BMBs) are associated with amyloid angiopathy and cognitive impairment, and with risk of dementia after intracerebral hemorrhage [9]. However, there is less information on the effect of BMBs on cognition after minor stroke [9]. Whilst a study reported BMBs, mainly in the frontal lobe and basal ganglia, being strongly associated with cognitive dysfunction, independently of the extent of WMH and the occurrence of a previous ischemic stroke [10], other studies have not found BMBs to be an independent determinant factor of post-stroke cognitive performance [11,12].

Prevalence and progression of striatal ID after stroke is also under-researched. A few animal studies have found a delay in iron sequestration in the brain due to dysfunctional brain iron regulatory mechanisms and continued damage to neuronal tissue following ischemic stroke [13,14], causing prolonged duration of iron neurotoxicity. A study in young human adults found increased ID in the basal ganglia, subcortical and periventricular white matter and thalami following infarcts [15]. Conflicting results have also been reported on the progression of the number of BMBs after stroke: whilst a study reported progression in 19% of stroke patients after a 2-year period [16], other studies have not found progression [17] or difference in the number of BMBs in stroke patients with respect to controls [18]. Inter-observer differences [19], and variations in magnetic field strengths and sequence parameters have been considered limitations in the assessment of BMB progression [20].

We aim, therefore, to test two hypotheses, that: (1) Striatal ID and BMB volumes increase from onset to 1 year after a mild-to-moderate ischemic stroke; and (2) Striatal IDs and BMBs accumulations are associated with worse cognition assessed 1 year after a mild-to-moderate ischemic stroke, accounting for vascular risk factors and age differences. We studied a cohort of small vessel disease (SVD) patients who had had a mild stroke.

## 2. Results

### 2.1. Sample Characteristics

**Figure 1:**
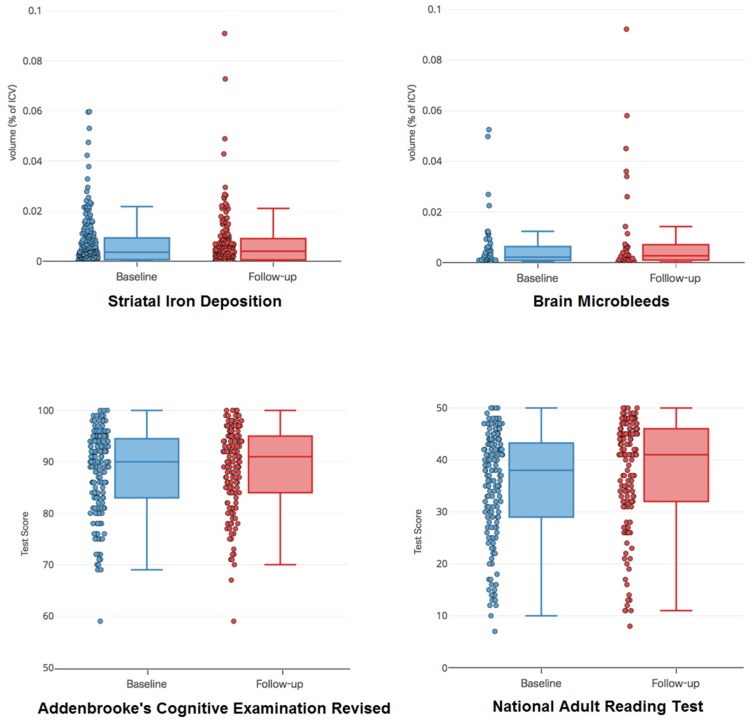
Box plots and distributions of the main imaging and cognitive variables analyzed: Striatal (mainly basal ganglia) iron deposition, brain microbleeds, Revised Addenbrooke’s Cognitive Examination, and the National Adult Reading Test.

**Figure A1:**
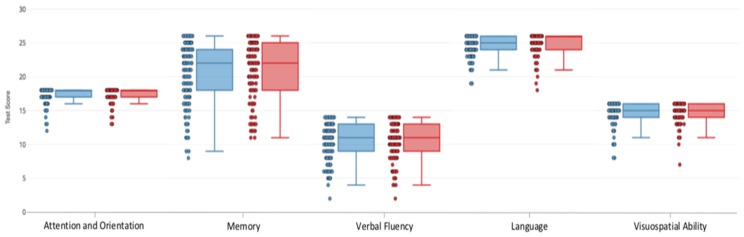
Box plots and distributions (values) of the ACE-R subcomponent scores at 1–3 months (blue) and 1 year (red).

The descriptive characteristics for the total sample at each time point are presented in Table 1 and Figure 1 and Figure A1. Basal ganglia IDs were identified in 209/264 (79%) patients at baseline and 152/190 (80%) patients at follow-up, and BMBs were identified in 58/264 (22%) patients at baseline and 42/190 (22%) patients at follow-up. 190 patients had the follow-up 1-year MRI scan, being the drop-out reasons previously published [21] (see analysis of missing values in the Supplementary Material Figures S1.25 to S1.27). The proportion of patients that had each vascular risk factor did not perceptively change between the two assessment waves. From the patients that had BMBs at baseline, only 14 had 5 or more BMBs, of which 9 had a follow-up scan. Other types of mineral deposition identified were residuals from petechial hemorrhages (6 patients), an aneurism (1 patient), and dural calcifications (2 patients). Patients presenting with lacunar stroke had, in proportion, the highest volume of BMBs in the sample, followed by the few patients who had a small cortical stroke in the brainstem (Table 2). The average volume of BMBs was higher in patients who had the index stroke in the left hemisphere compared to those who had the stroke in the right hemisphere (Table 2). Extended information of the spatial distribution (per arterial territory and hemisphere) of the different stroke lesion clusters (new and old) in patients with and without BMBs at baseline and after a year can be found in the Supplementary Material (Table S2.1, Figures S2.1 and S2.2).

Cognitive data were obtained from 157 patients at 1-3 months and 151 patients at 1 year. Table A2 shows the demographics and stroke characteristics of the subsample that provided cognitive data at each time point, and the drop-out reasons. The patients who had cognitive testing were younger than those who were not tested (mean age 66 *vs.* 71 respectively), but were no different in terms of NIHSS score (i.e., NIHSS in both groups was 2 (IQR 1-3)) or recurrent stroke (12/151 and 5/57 patients respectively) during the follow-up period.

### 2.2. Progression of Imaging Markers and Cognitive Scores

Considering only the subsample that had follow-up MRI scans (i.e., 190 patients), the volume of basal ganglia IDs (expressed as percentage in ICV) did not significantly change over the one year period (median difference: 0.0003 IQR [0; 0.0011] % in ICV volume, *p* = 0.718). From this subsample (i.e., patients that were scanned at baseline and 1-year after), the BMB volumes in the patients that had baseline BMBs slightly decreased after a year, but this decrease was not significant (median difference: −0.0003 IQR [−0.00009; −0.0011] % in ICV volume, *p* = 0.637). The patients that did not have a BMB at baseline did not have them at the follow-up either (147/190). From the 9 patients that had ≥ 5BMBs at baseline, 8 had higher BMB volume a year afterwards.

From 135 patients who underwent cognitive testing both at 1–3 months and 1-year, 6 did not have MRI data at both time points, so our final longitudinal sample with both MRI-derived measurements and cognitive scores was 129 patients. Table A1 provides the descriptive statistics for the subsample that provided data at both time points. The median ACE-R score did not change between assessments, however NART scores increased from median [IQR] = 38 [30; 43] at 1–3 months to 41 [32; 46] at 1-year (*p* = 0.001) (Table A1).

### 2.3. Bivariate Relations

**Figure 2:**
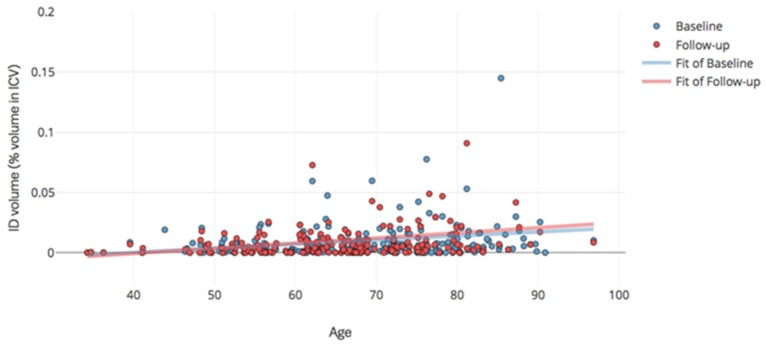
Correlation between age and volume of striatal iron deposition at both: baseline (blue) and follow-up (red).

Non-parametric (Spearman ρ) correlations (with and without bootstrap with *n* = 1000) between age, gender, imaging and cognitive variables at both time-points are presented in Table 3. As expected, baseline ID and BMB volumes were positively correlated (Spearman ρ = 0.238, *p* < 0.0001). Follow-up ID and BMB volumes were also positively correlated (Spearman ρ = 0.204 *p* = 0.005). ID volumes correlated with NART scores at both time points (Spearman ρ=0.198 *p* = 0.014 and Spearman ρ = 0.182 *p* = 0.036 respectively). Total baseline ACE-R scores were not correlated with any imaging variable. Baseline WMH volumes correlated with ID and BMB volumes at baseline and follow-up (Table 3). Age correlated with BGID volumes at both time points (ρ = 0.248, *p* < 0.001 and ρ = 0.271, *p* < 0.001 respectively, Table 3, Figure 2) but with BMB volume only at baseline (ρ = 0.129, *p* < 0.05); and also with ACE-R but not with NART. Bootstrap and adjusting for brain tissue volume (instead of head size) did not change the pattern of the correlations.

### 2.4. Association Between Baseline Striatal Iron Deposition and 1-year Cognition

After accounting for vascular risk factors, age, sex, WMH volume, and cognition at 1–3 months post-stroke, baseline striatal ID was not associated with 1-year cognitive scores or with their change from 1–3 months to a year (i.e., with change defined as the cognitive score at 1 year minus the score at 1–3 months). However, in the same models, smoking and baseline WMH burden predicted verbal fluency and visuospatial ability scores a year after the stroke, respectively (Table 4). Results did not differ with models that used 1-year ACE-R scores as an outcome variable, which accounted for premorbid intelligence (i.e., 1–3 months NART).

### 2.5. Association Between Baseline Brain Microbleeds and 1-year Cognition

Baseline volume of BMBs was only associated with the change in the ACE-R visuospatial scores from 1–3 months to 1 year, but with borderline significance. In these models smoking and baseline WMH burden were predictors of the verbal fluency and visuospatial abilities scores (respectively) a year after the stroke (Table 4). Results did not differ when models accounted for premorbid intelligence (i.e., 1–3 months NART).

**Figure 3:**
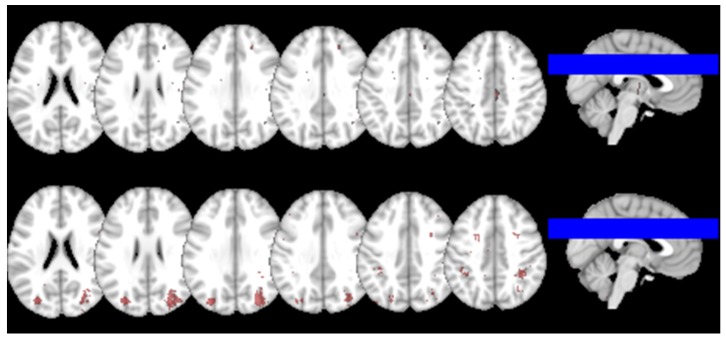
Probability Distribution Map of the BMBs in this sample (above) and Reverse Inference Map of the brain regions that were preferentially related to the term visuospatial in the 224 studies (below). The locations that show the red-to-white foci appear to be reported more often in articles that include the term visuospatial in their abstracts than in articles that do not.

We investigated whether our finding was in-line with the published literature searching in the meta-analyses brain database Neurosynth (http://www.neurosynth.org/) for neural correlates of the outcome of visuospatial and reading tests. The search resulted in 224/11406 studies in the database for the term visuospatial. The reverse inference map resulting from the meta-analysis of the selected publications, after false discovery rate correction (expected FDR 0.01 as per website documentation) for the term visuospatial is shown in Figure 3 (bottom row). The same figure also shows the probability distribution map of BMBs (upper row). As can be appreciated, there is only a modest overlap with the occurrence of BMBs in our cohort in the regions that have been shown to be related to the term visuospatial in the studies included in this database.

### 2.6. Risk Factors for ID and BMB Progression

Only the change in the volume of BMBs (adjusted by head size) had a weak borderline association with gender (B = 0.00005, *p* = 0.045). None of the vascular risk factors evaluated were associated with the volumes of striatal IDs or BMBs a year after the stroke after accounting for baseline volumes.

## 3. Discussion

### 3.1. Progression Pattern of IDs and BMBs Following a non-Disabling Ischemic Stroke

Contrary to our hypothesis, there was no progression of BGID volume or BMBs 1 year following a mild-to-moderate ischemic stroke. BGID are known to accumulate with age [1,3,4], and we found a positive correlation between BGID volumes and age, confirmatory of the body of literature on the theme. However, the speed of the volumetric increase of these mineral deposits in tissue, and the factors that might accelerate it (albeit metabolic [5]) are not known. A duration of one year in a relatively small sample with a wide age range might not be sufficient to confidently detect the change that was observed but which could have occurred by chance (i.e., statistically not significant). BMBs, specifically, were infrequent, present in only a small proportion of individuals from each time point (58 patients at baseline; 42 at follow-up); and BMB prevalence (22%) remained stable from baseline to 1 year after stroke. It is difficult to assert whether our findings differ or don’t differ from reports in the current literature. Lee et al. (2011) [22], for example, examined 224 stroke patients for over 3 years and described an overall incidence of 0.8 new BMBs per year, increasing to 5.4 per year in patients with ≥5 BMBs at baseline. However, we only analyzed the progression in one year on a sample where only 9 patients had ≥5 BMBs at baseline. Brain iron appearance on MR images varies depending on the time and aggregation of these particles in tissue [23,24]. Of the ischemic stroke subtypes, BMBs have reported to occur and increase more frequently in patients with lacunar infarcts (26–62%) than cardio-embolic (4–30%) or atherothrombotic infarcts (21–46%) [25,26,27,28,29,30]. Indeed, an increase in the volume of BMBs was observed in all (except one) of the patients of our study with ≥5 BMBs at baseline, and which had lacunar stroke, but we can’t draw strong conclusions from the analysis of only nine patients. With regards to the progression of basal ganglia ID volume, there was no change. The complexities measuring mineral deposition on conventional structural gradient echo and/or susceptibility-weighted images have been widely discussed previously [31]. Our results warrant further confirmation in future studies.

### 3.2. ID and BMB Volumes as Predictors of 1-year Cognition following Ischaemic Stroke

Striatal ID volume was correlated with NART, which represents premorbid IQ, at both baseline and 1 year post-stroke, confirming that this type of brain mineral accumulation is not only a biomarker of general cognitive ability in middle-to-late adulthood [1], but that it is also related to the lifelong-stable trait of intelligence [3]. However, baseline striatal ID volume was not predictive of 1-year post-stroke cognitive abilities after accounting for 1–3 months post-stroke cognitive abilities or NART. Fluctuations in cognition shortly following stroke have been reported previously [32], with improvements particularly at 1 year [32,33,34]. A previous study on post-stroke long-term cognition found that despite NART performance being worse at 1–3 month post-stroke compared to 1 year, the overall average NART performance 3 years after the stroke was even lower than at 1-3 months post-stroke [34]. It is possible that the 1–3 month NART scores may be affected by short-term speech disturbances.

Striatal ID and BMB volumes were correlated at both baseline and 1 year post-stroke. The latter were not correlated or associated with cognitive measures at any time point. In general, the association between striatal ID volume, number of BMBs and cognitive functions have been reported previously [2,3,10,35,36]. Studies in patients with cerebral small vessel disease [10,36] and following an ischemic stroke [37,38] have reported associations between BMBs and cognitive dysfunction, as well as studies in normal adults [39,40] and elderly populations [41]. It has been suggested that subcortical networks may be severely disrupted due to the presence of BMBs [10]. However, in general, brain atrophy (i.e., global and regional with variations), stoke location and severity, and WMH burden, and not BMBs, have been considered to be the key factors impacting post-stroke cognition [42,43,44,45].

### 3.3. Risk Factors for ID and BMB Progression following Ischaemic Stroke

Vascular risk factors did not predict ID or BMB burden evolution 1-year post-stroke. The prevalence of IDs and BMBs has been associated with vascular risk factors [46]. For example, studies have found BMB prevalence to be associated with low total cholesterol [41,47], hypertension [41], and hyperlipidemia [48]. Risk factors for increased iron depositions have been reported to be similar to those for prevalent BMBs [46]. Arterial hypertensive patients have been found to have higher subcortical iron content in brain tissue than normotensive adults [49,50,51]. However, the influence of vascular risk factors in the progression of these forms of IDs has been less well documented. In a longitudinal study of ageing, subclinical elevations in a compound index of vascular risk factors predicted greater iron content in the putamen, but not in the caudate or hippocampus [52].

The mechanisms explaining the association between cardiovascular health and regional brain iron accumulation are unclear. They may plausibly reflect reduced cerebral blood flow [46] that would slow the delivery of iron-binding complexes to the brain [53]. Global decrease in cerebral blood flow may also decrease the function of the brain vascular endothelium in regulating iron uptake and clearance at the blood-brain barrier [54]. These cardiovascular and metabolic risk factors could, consequently, influence cognition by increasing the accumulation of IDs and iron deposition from BMBs following a non-disabling ischemic stroke, if such an increase is observed following the stroke event and it proves to have an effect on cognition. Alternatively, IDs could just be markers of impaired small vessel function which itself might be the cause of cognitive impairment. In our sample, in the period evaluated, an increase in the volume of IDs was not observed. Moreover, the size of the subsample that had BMBs and IDs was relatively small compared to samples used by other studies [41,47,48,55]. ID or BMB prevalence are determined by study sample characteristics. Finally, in our sample, the mean age was 65-67 and ID/BMB prevalence and number is known to increase with age [48].

### 3.4. Strengths and Limitations

This research adds to the existent body of literature investigating the role IDs and BMBs play on cognition, and their longitudinal association with cognitive decline following a non-disabling ischemic stroke, being explored here for the first time. Amongst its strengths are the use of state-of-the-art image processing methods to assess ID/BMB volumes, performed blind to any clinical, cognitive and demographic data, the assessment under the same MRI protocol of a large cohort of individuals using well-established guidelines, and the extensive assortment of available relevant participant data. Although the volumetric measurements of iron accumulation in this analysis are as accurate as they could possibly be, structural MRI techniques cannot accurately determine the actual volume of iron accumulation in brain tissue [1]. The volumetric measurements, therefore, rather reflect the effect iron particles have on the magnetic resonance (MR) signal. The metal/metalloid particles’ susceptibility is influenced by their proportions, aggregations and interactions with underlying tissues, all of which further effect the MR signal [4]. Another limitation is that it was only possible to obtain valid quantitative measures of IDs, BMBs and cognition scores from 44% of the available dataset; this was mainly due to patients not attending follow-up sessions or not completing cognitive testing at both assessment waves. Thus, the regression analyses ended-up using data from ~60% fewer individuals, which were healthier than the dropouts. The original sample size calculation was based on assumed differences in small vessel disease progression between cortical and lacunar stroke patients at one year post recruitment, and therefore there is no sample size calculation for the relationship between iron deposits, microbleeds, and cognition. At the time of recruitment, there would have been no previous estimates on which to base a sample size calculation. We have not performed a post-hoc power calculation as these are flawed and are not recommended [56]. We have reported all analyses transparently so that readers can judge the validity for themselves. It is emphasized that given this is a cohort of participants with minor stroke, the findings will relate to this group rather than community dwelling healthy individuals. Moreover, in this study, the location of BMBs was observed but was not statistically analyzed. Previous research has identified associations between lobar BMBs and longitudinal cognitive decline, whereas no associations were revealed between BMBs located in the infratentorial region and the deep grey matter of the brain [57]. As BMBs from all regions were included in this study, the results could have potentially been influenced and the findings diluted. Lastly, although the fully automatic nature of these ID/BMB measures makes them robust against inter-/intra-observer variations, the essential manual post-processing corrections made to all multifocal T2*W hypointensities are susceptible to observer variations [19].

### 3.5. Future Work

Future studies should seek to increase the duration of the study and account for inter-observer differences in the statistical models. The analysis of IDs/BMBs could be performed fully computationally, however, higher spatial resolution and perhaps imaging on higher afield strengths would be needed. In addition, quantitative imaging or phase imaging would be desirable for computationally assessing these imaging features. Different results may be yielded by repeating this study in a few years-time. ID volume could potentially have a more pronounced effect on cognitive measures as the sample population ages. Similar study on a larger cohort of patients could also be performed. Analyzing iron load in thalamic tissue and in the substantia nigra may also be informative. Useful information is likely to be provided by such study on the progression of iron depositions in these regions associated risk factors, confirming or otherwise the potential valuable role, following an ischemic stroke, of IDs as predictors or indicators of cognitive decline.

## 4. Materials and Methods

### 4.1. Subjects

The sample was composed of 264 patients (154 men, 110 women) obtained from a study of SVD and stroke mechanisms [21]. Briefly, patients were aged ≥18 years and had a diagnosis of lacunar or mild cortical ischemic stroke. They were excluded if they lacked capacity to consent for participating in the study, had concomitant serious medical disorders making clinical follow-up unlikely or impossible (e.g., disabling stroke), had any contraindication to MRI scan, showed an alternative diagnosis at initial MRI scan (e.g., multiple sclerosis, cancer), or had severe renal impairment. Full recruitment and assessments have been previously published [21,58]. Written informed consent was obtained from all patients on protocols approved by the Lothian Ethics of Medical Research Committee (REC 09/81101/54) and NHS Lothian R + D Office (2009/W/NEU/14), on the 29 October 2009.

### 4.2. Clinical Data

We used the following clinical data selected based on clinical plausibility and/or previous research of stroke, collected at diagnosis and determined following the criteria given in the primary study [21]: hypertension, hyperlipidemia, and smoker status (i.e., current or recent smoker *vs.* no-smoker or ex-smoker (i.e., more than 1 year)).

### 4.3. MRI Acquisition

We performed MRI at diagnosis and one-year after index stroke on a GE Signa Horizon HDx 1.5T clinical scanner (General Electric, Milwaukee, WI, USA) equipped with a self-shielding gradient set (33 mT/m maximum gradient strength) and manufacturer supplied 8-channel phased-array head coil, following identical imaging protocols at both time points. The primary sequence used to identify IDs and BMBs was T2*-weighted (T2*W) gradient echo sequence acquired in axial orientation, with TE/TR = 15/800, flip angle 20º, and with an in-plane resolution of 384 × 224 voxels. We also used the T1-weighted (T1W) structural sequence that acquired 3D in sagittal orientation, with an inversion recovery-prepared spoiled gradient echo (SPGR) (TR/TE/TI = 7.3/2.9/500 ms, 8° flip angle, 330 × 214.5 cm FoV, 256 × 146 acquisition matrix, 100 × 1.8 mm slices). The full imaging protocol is described in reference [58].

### 4.4. Image Analysis

We assessed multifocal T2*W hypointensities in the basal ganglia fully automatically using the method described in reference [31], available from https://github.com/aglatz/mineral-deposit-segmentation-pipeline/tree/master/libBRIC/mineral-deposit-segmentation. This pipeline uses atlas-based tools to extract the region of interest in which the segmentation algorithm performs, namely the basal ganglia and internal capsule. We visually checked all segmentations for accuracy, and manually corrected where necessary (i.e., approximately 33% of cases) using Mango (Multi-image ANalyses Graphic unit interface, http://ric.uthscsa.edu/mango/). We segmented BMBs and other types of brain mineral deposition (e.g., hemorrhages, calcifications) elsewhere semi-automatically following validated threshold-based in-house guidelines using the same software. All assessments were done blind to any demographic, clinical or cognitive data. We obtained white matter hyperintensity volumes (reported in reference [21]) from the primary study database. These are found to play a mediating role in the effect that IDs have in cognition [2]. We adjusted all volumetric measurements by intracranial volume (ICV), also obtained from the primary study database. We also visually rated white matter hyperintensities (WMH) using Fazekas scores, basal ganglia IDs [3,59] and BMBs [9] to cross-validate the computational measures.

### 4.5. Cognitive Assessments

We administered the Revised Addenbrooke’s Cognitive Examination (ACE-R, [60]) and the National Adult Reading Test (NART, [61]) at one-to-three months and one year after the index stroke. Both tests were scored in accordance with the scoring guidelines.

### 4.6. Statistical Analyses

We used MATLAB R2017b (https://es.mathworks.com) for our statistical analyses. We used the paired samples Wilcoxon signed rank test to assess differences between IDs and cognitive variables at both time points, and the Spearman’s rank-order correlation with bootstrap as implemented in the Robust Correlation Toolbox [62] to explore the strength and direction of the bivariate monotonic relationships between cognitive and imaging variables at both time points. Cases were excluded pairwise in these tests. Results were double-checked using SPSS Statistics 21.0.0.

We analyzed data distribution and missing values (Supplementary Material, Tables and Figures from S1.1 to S1.24). ACE-R attention and orientation, ACE-R visuospatial abilities, and ACE-R language were transformed. We used multinomial logistic regression models to explore the putative association between baseline ID measurements and the outcome of these cognitive tests at 1 year. We used ANCOVA models to explore the associations between baseline striatal ID and BMBs, and the results of NART, total ACE-R, ACE-R memory and ACE-R verbal fluency; and analyze possible predictors for potential change in IDs and BMBs volumes. Covariates in all models were age, gender, baseline WMH volume and vascular risk factors (i.e., hypertension, hyperlipidemia, and smoker status) were selected a priori based on clinical plausibility and/or previous research. In each model, baseline ID and BMB volumes were adjusted for cognition at 1–3 months post-stroke (i.e., ACE-R test score or NART depending on the outcome variable). The analysis was repeated adjusting by 1–3 months NART in all cases, as this test is considered a surrogate for assessing premorbid intelligence [34]. BMBs were always analyzed separately from the striatal IDs, as their etiologies differ. Cases were excluded listwise. Also, the striatal ID and BMB volumes were standardized to the ICV to derive the percentage of IDs and BMBs in ICV.

## 5. Conclusions

BGIDs and BMBs are associated differently with cognition post-stroke; studies of imaging and post-stroke cognition should adjust for premorbid cognition. The positive correlation of BGID with NART is likely to reflect the lower premorbid cognition in patients with stroke at younger vs older ages.

## Figures and Tables

**Table 1:** Descriptive statistics of the total sample at diagnosis/first wave of cognitive testing, and 1 year after (follow-up). For simplification of tabular presentation, percentages (%) are rounded to the closest integer number (elsewhere, test percentages are shown up to two significant decimal places).

		*Baseline Measurements*	*1 Year*
*Variable Types*		(*n* = 264)	(*n* = 190)
Age (years) [mean (SD)]		67 (11.84)	65 (11.28)
Gender [% (n)]			
	Male	58 (154)	58 (111)
	Female	42 (110)	42 (79)
*Brain Measurements*			
	Lacunar stroke [% (n)]	45 (118)	46 (88)
	Cortical stroke [% (n)]	55 (146)	54 (102)
	ICV (ml) [mean (SD)]	1478.33(146.47)	1479.81 (147.85)
	WMH (%ICV) [median (IQR)]	0.89 (0.31–2.39)	0.98 (0.42–2.16)
	ID [% (n)]	79 (209)	80 (152)
	ID (%ICV) [median (IQR)]	0.0039 (0.00060–0.0099)	0.0043 (0.00057–0.010)
	BMB [% (n)]	22 (58)	22 (42)
	BMB (%ICV) [median (IQR)]†	0.0019 (0.00076–0.0049)	0.0019 (0.00061–0.0038)
	Haemorrhage [% (n)]	2 (6)	3 (6)
	Haemorrhage (%ICV) [median (IQR)]†	0.014 (0.0091 - 0.048)	0.036 (0.011–0.054)
*Cognitive Test Scores [median (IQR)]*		*(*n* = 157)	*(*n* = 151)
	ACE-R Total	90 (83–94)	91 (84.75–95)
	ACE-R Attention & Orientation	18 (17–18)	18 (17–18)
	ACE-R Memory	22 (18–24)	22.5 (18–25)
	ACE-R Verbal Fluency	11 (9–13)	11 (9–13)
	ACE-R Language	25 (24–26)	26 (24.75–26)
	ACE-R Visuospatial Ability	15 (14–16)	15 (14–16)
	NART Total	37.5 (29–43)	41 (32–46)
*Past Medical History [% (n)]*			
	Hypertension	72 (191)	74 (141)
	Hyperlipidaemia	61 (161)	61 (116)
	Current smoker	34 (90)	34 (65)
	Recent ex-smoker	5 (12)	4 (8)
	Ex-smoker	28 (74)	25 (48)
	Never smoker	33 (87)	36 (69)

Note: *(n): Sample size where data was unavailable. Demographics of these subsamples are in Appendix Table A2. †: Calculated only in patients that had the feature.

**Table 2:** Brain microbleed volume and number of patients with vs. without microbleeds per index stroke lesion subtype, cerebral hemisphere and arterial territory.

Index Stroke Lesion Subtype, Arterial Territory and Cerebral Hemisphere	No. of Patients without BMB	No. of Patients with at Least 1 BMB	Average Volume of BMB Expressed as % in ICV (SD)	Total no. of Patients (%)
Cortical in the Middle Cerebral Artery (MCA) territory	43	8	0.00059 (0.0017)	51 (19.3)
Cortical in the Anterior Cerebral Artery (ACA) territory	4	0	0	4 (1.5)
Cortical in the Posterior Cerebral Artery (PCA) territory	20	5	0.00058 (0.0019)	25 (9.5)
Cortical in the border zone (i.e., watershed) territories	18	5	0.00055 (0.0015)	23 (8.7)
Lacunar	47	28	0.0017 (0.0038)	75 (28.4)
Cortical in Cerebellum	7	0	0	7 (2.7)
Cortical in Brainstem	1	3	0.0012 (0.0013)	4 (1.5)
Ischemic stroke in Right Hemisphere	81	23	0.00064 (0.0017)	104 (39.4)
Ischemic stroke in Left Hemisphere	59	26	0.0014 (0.0035)	85 (32.2)

**Table 3:** Non-parametric bivariate correlations between the imaging and main cognitive variables evaluated, age and gender; with bootstrap (upper right hand side triangle) and without bootstrap (bottom left hand side triangle). analyzed Correlations between all variables (see legend) were analyzed using Spearman’s rank-order correlation. Sample numbers of each variable differed and are reported in the descriptive characteristics of the data sample (Table 1). Spearman (ρ) values and indication of the significance level: * *p* < 0.05, ** *p* < 0.001 are given.

	(1)	(2)	(3)	(4)	(5)	(6)	(7)	(8)	(9)	(10)	(11)
**(1) Age**	1	0.009	***0.443*** **	***0.296*** **	***0.209*** *	**−*0.305*** **	0.074	***0.297*** **	***0.184*** *	−0.242 **	0.109
**(2) Gender**	−0.043	1	0.131	0.177	0.016	0.051	***0.246*** **	***0.227*** *	0.042	0.081	0.148
**(3) Baseline WMH volume (% in ICV)**	***0.506*** **	0.011	1	***0.377*** **	***0.292*** **	−0.143	***0.225*** *	***0.408*** **	***0.306*** **	−0.155	0.135
**(4) Baseline BGID volume (% in ICV)**	***0.248*** **	0.031	***0.279*** **	1	0.156	0.040	***0.219*** *	***0.799*** **	0.171	0.024	0.194 *
**(5) Baseline BMB volume (% in ICV)**	***0.129*** *	0.065	***0.294*** **	***0.238*** **	1	−0.100	0.092	0.179	***0.977*** **	−0.055	0.039
**(6) Baseline ACE-R**	**−*0.322*** **	0.038	−0.131	−0.023	-0.104	1	***0.462*** **	0.018	−0.081	0.758 **	0.470 **
**(7) Baseline NART**	0.087	***0.202*** *	***0.204*** *	***0.198*** *	0.071	***0.468*** **	1	***0.270*** **	0.117	0.535 **	0.866 **
**(8) Follow-up BGID vol. (% in ICV)**	***0.271*** **	***0.146*** *	***0.367*** **	***0.835*** **	***0.192*** **	−0.028	***0.241*** **	1	***0.200*** *	0.033	0.219 *
**(9) Follow-up BMB vol. (% in ICV)**	0.142	0.068	***0.309*** **	***0.191*** **	***0.985*** **	−0.126	0.071	***0.204*** **	1	−0.037	0.057
**(10) Follow-up ACE-R**	**−*0.241*** **	0.091	**−*0.177*** *	−0.008	−0.057	***0.788*** **	***0.526*** **	−0.014	−0.053	1	0.559 **
**(11) Follow-up NART**	0.113	0.138	0.092	0.132	0.011	***0.477*** **	***0.854*** **	***0.182*** *	0.032	0.568 **	1

**Table 4:** Results from the models that explore effect of baseline mineral deposition in the striatum, and, separately, brain microbleeds in cognition 1 year after the stroke, accounting for cognition 1–3 months after the stroke. All models used age, gender, vascular risk factors, and baseline white matter hyperintensity volume as covariates. The association (B) and standard error (SE) from all terms of the models are given. p-values are only given if significant (*p* < 0.05).

Outcome Variable (Dependent)	Predictor (Independent Variable)	Main Effect (B, (SE))	Covariates					
			Age	Gender	Hypertension	Hyper-lipidemia	Smoking	% Baseline WMH vol. in ICV
***Follow-up ACER***	**Baseline % striatal ID vol. in ICV**	−17.35 (11.60)	−0.0081 (0.044)	0.30 (0.84)	0.72 (0.95)	0.54 (0.89)	−1.38 (0.89)	−0.091 (0.32)
	Baseline % BMB vol. in ICV	130.32 (215.68)	−0.018 (0.045)	0.43 (0.85)	0.76 (0.96)	0.49 (0.91)	−1.25 (0.90)	−0.23 (0.34)
***ACE-R change***	Baseline % striatal ID vol. in ICV	−17.36 (11.60)	−0.0081 (0.044)	0.30 (0.84)	0.72 (0.95)	0.54 (0.89)	−1.38 (0.89)	−0.091 (0.32)
	Baseline % BMB vol. in ICV	130.32 (215.68)	−0.018 (0.045)	0.32 (0.85)	0.75 (0.96)	0.49 (0.91)	−1.25 (0.90)	−0.23 (0.34)
***Follow-up Orientation (†)***	Baseline % striatal ID vol. in ICV	−2.25 (7.52)	0.02 (0.02)	−0.10 (0.43)	−0.60 (0.46)	−0.33 (0.43)	0.083 (0.46)	0.15 (0.16)
	Baseline % BMB vol. in ICV	−18.45 (103.11)	0.024 (0.023)	0.21 (0.46)	−0.64 (0.50)	−0.50 (0.48)	0.014 (0.49)	0.17 (0.18)
***Orientation change***	Baseline % striatal ID vol. in ICV	0.46 (1.16)	−0.0045 (0.0043)	−0.029 (0.084)	0.12 (0.094)	0.094 (0.089)	−0.0025 (0.089)	−0.035 (0.032)
	Baseline % BMB vol. in ICV	2.26 (21.38)	−0.0045 (0.0043)	−0.035 (0.084)	0.12 (0.095)	0.095 (0.090)	−0.0042 (0.090)	−0.033 (0.034)
***Follow-up Memory***	Baseline % striatal ID vol. in ICV	−12.79 (8.05)	−0.023 (0.030)	−0.10 (0.58)	0.63 (0.67)	0.74 (0.62)	−0.22 (0.62)	0.095 (0.22)
	Baseline % BMB vol. in ICV	66.67 (150.32)	−0.029 (0.031)	−0.055 (0.59)	0.66 (0.68)	0.71 (0.63)	−0.14 (0.63)	0.0015 (0.23)
***Memory change***	Baseline % striatal ID vol. in ICV	−12.793 (8.05)	−0.023 (0.031)	−0.10 (0.58)	0.63 (0.67)	0.74 (0.62)	−0.22 (0.62)	0.095 (0.22)
	Baseline % BMB vol. in ICV	66.67 (150.32)	−0.029 (0.031)	−0.055 (0.59)	0.66 (0.68)	0.71 (0.63)	−0.14 (0.63)	0.0015 (0.23)
***Follow-up Verbal Fluency***	***Baseline % striatal ID vol. in ICV***	−8.00 (5.26)	−0.0091 (0.020)	0.39 (0.38)	0.43 (0.43)	−0.14 (0.40)	**−1.13 (0.41)** **(*p* = 0.0067)**	0.12 (0.15)
	***Baseline % BMB vol. in ICV***	7.62 (97.81)	−0.012 (0.020)	0.41 (0.39)	0.41 (0.43)	−0.19 (0.41)	−**1.06 (0.41)** **(*p* = 0.011)**	0.08 (0.16)
***Verbal Fluency change***	***Baseline % striatal ID vol. in ICV***	−8.01 (5.26)	−0.0091 (0.020)	0.39 (0.38)	0.43 (0.43)	−0.14 (0.40)	**−1.13 (0.41**) **(*p* = 0.0067)**	0.12 (0.15)
	***Baseline % BMB vol. in ICV***	7.62 (97.81)	−0.012 (0.020)	0.41 (0.39)	0.41 (0.43)	−0.19 (0.41)	−1.06 (0.41) (*p* = 0.011)	0.08 (0.16)
***Follow-up Language (†)***	Baseline % striatal ID vol. in ICV	−14.058 (13.88)	0.01 (0.02)	-0.048 (0.45)	0.39 (0.48)	0.53 (0.45)	−0.77 (0.48)	0.079 (0.16)
	Baseline % BMB vol. in ICV	−39.90 (123.038)	0.014 (0.023)	0.11 (0.47)	0.51 (0.52)	0.32 (0.49)	−0.77 (0.50)	0.072 (0.18)
***Language change***	Baseline % striatal ID vol. in ICV	2.41 (2.35)	−0.0062 (0.0088)	0.13 (0.18)	−0.13 (0.19)	−0.21 (0.18)	0.15 (0.18)	−0.034 (0.065)
	Baseline % BMB vol. in ICV	−19.45 (46.74)	−0.0055 (0.0096)	0.050 (0.19)	−0.064 (0.21)	−0.23 (0.19)	0.14 (0.20)	−0.013 (0.073)
***Follow-up Visuospatial (†)***	Baseline % striatal ID vol. in ICV	2.93 (10.10)	0.019 (0.022)	−0.62 (0.41)	0.37 (0.45)	−0.18 (0.42)	1.00 (0.45)	0.19 (0.16)
	Baseline % BMB vol. in ICV	−110.92 (118.39)	0.022 (0.023)	−0.75 (0.44)	0.65 (0.50)	−0.52 (0.47)	0.88 (0.47)	0.36 (0.19)
***Visuospatial change***	***Baseline % striatal ID vol. in ICV***	1.44 (2.41)	−0.0018 (0.0091)	0.27 (0.18)	−0.22 (0.20)	0.12 (0.19)	−0.29 (0.19)	**−0.22 (0.067) (*p* = 0.0015)**
	***Baseline % BMB vol. in ICV***	**90.34 (45.56)** **(*p* = 0.05)**	−0.0045 (0.0094)	0.11(0.17)	−0.15 (0.20)	0.11 (0.19)	−0.33 (0.19)	**−0.21 (0.071) (*p* = 0.0044)**
***Follow-up NART***	Baseline % striatal ID vol. in ICV	6.92 (14.50)	0.030 (0.054)	-0.51(1.07)	0.33 (1.18)	−1.86 (1.12)	−0.88 (1.12)	−0.59 (0.41)
	*Baseline % BMB vol. in ICV*	86.72 (268.56)	0.020 (0.054)	−0.57 (1.075)	0.39 (1.20)	−1.79 (1.13)	−0.94 (1.13)	−0.60 (0.43)
***NART change***	Baseline % striatal ID vol. in ICV	6.92 (14.50)	0.030 (0.054)	−0.51 (1.07)	0.33 (1.18)	−1.86 (1.12)	−0.88 (1.12)	−0.59 (0.41)
	*Baseline % BMB vol. in ICV*	86.72 (268.56)	0.020 (0.054)	−0.57 (1.075)	0.39 (1.20)	−1.79 (1.13)	−0.94 (1.13)	−0.60 (0.43)

Legend: WMH: white matter hyperintensities, ID: iron deposition, BMB: brain microbleeds, ACE-R: Addenbrooke’s Cognitive Examination Revised, NART: National Adult Reading Test, (†): Dichotomised cognitive variables used multinomial logistic regression models (see text)).

## Supplementary Materials

Supplementary materials can be found at https://www.mdpi.com/1422-0067/20/6/1293/s1.

## Abbreviations

BGID	Basal Ganglia Iron Deposition
BMBs	Brain MicroBleeds
NART	National Adult Reading Test
ACE-R	Addenbrooke’s Cognitive Examination - Revised
WMH	White Matter Hyperintensities
MRI	Magnetic Resonance Imaging
IDs	Iron Deposits
ICV	IntraCranial Volume
IQR	Inter Quartile Range
ANCOVA	ANalysis of COVAriances

## Appendix A

**Table A1:** Descriptive statistics of the subsample used in the ANCOVA analyses (i.e., patients with complete ID measurements and cognitive test results at diagnosis/first wave of cognitive testing and 1 year after) (*n* = 129). For simplification of tabular presentation, percentages (%) are rounded to the closest integer number (elsewhere, test percentages are shown up to two significant decimal places).

		*Baseline measurements*	*1 Year*
*Variable Types*			
Age (years) [mean (SD)]		64.47 (10.58)	
Gender [% (n)]			
	Male	65 (84)	
	Female	36 (46)	
*Brain Measurements*			
	Lacunar stroke [% (n)]	43 (55)	
	Cortical stroke [% (n)]	58 (75)	
	White Matter Lesion (%ICV) [median (IQR)]	0.76 (0.26–2.04)	0.95 (0.42–1.91)
	ID [% (n)]	77 (99)	76 (98)
	ID (%ICV) [median (IQR)]†	0.0051 (0.0025–0.011)	0.0059 (0.0032–0.011)
	BMB [% (n)]	22 (28)	21 (27)
	BMB (%ICV) [median (IQR)]†	0.0018 (0.00072–0.0051)	0.0016 (0.00071–0.0042)
	Haemorrhage [% (n)]	5 (6)	5 (6)
	Haemorrhage (%ICV) [median (IQR)]†	0.031 (0.012–0.14)	0.045 (0.019–0.083)
*Cognitive Test Scores [median (IQR)]*			
	ACE-R Total	91 (84–95)	91 (85–95)
	ACE-R Attention & Orientation	18 (17–18)	18 (17–18)
	ACE-R Memory	22 (18–25)	23 (19–25)
	ACE-R Verbal Fluency	11 (9–13)	11 (9–13)
	ACE-R Language	25 (24–26)	26 (25–26)
	ACE-R Visuospatial Ability	15 (15–16)	15 (14–16)
	NART Total	38 (30–43)	41 (32–46)
*Past Medical History [% (n)]*			
	Hypertension	73 (94)	
	Hyperlipidaemia	64 (83)	
	Current smoker	31 (40)	
	Recent ex-smoker	4 (5)	
	Ex-smoker	28 (36)	
	Never smoker	37 (48)	

†: Calculated only in the subsample that has the imaging feature.

**Table A2:** Demographics of patients who had cognitive testing vs. those recruited but who did not undergo cognitive testing.

	*Patients Tested*	*Patients Not Tested*	*p-value*
*1–3 Months*	*n* = 157 *	*n* = 51	
Age at index stroke (IQR)	66 (56–75)	71 (63–80)	<0.01
Female gender	64 (41%)	24 (47%)	0.51
Previous stroke (prior to index event)	19 (12%)	4 (10%)	0.8
*1 Year*	*n* = 151	*n* = 57	
Age at stroke (IQR)	66 (56–74)	73 (61–82)	<0.01
Female gender	58 (39%)	30 (52%)	0.51
Previous stroke (prior to index event)	19 (13%)	5 (7%)	0.58
Stroke during follow-up	12 (8%)	5 (9%)	0.78
*Cognition tested at 1–3 months, but not 1 year n = 22*			
Age at stroke (IQR)	65 (56–72.5)		
Female gender	12 (55%)		
Stroke during follow-up	2 (9%)		
Reasons not tested at 1 year	Declined repeat test 11, too unwell 10, deceased 1		
*Cognition not tested at 1–3 months, but tested at 1 year n = 16*			
Age at stroke (IQR)	72 (66–79.25)		
Female gender	6 (38%)		
Stroke during follow-up	1 (6%)		
Reasons not tested at 1–3 months	Dysphasia which improved 1, forgot reading glasses 2, unable to attend due to work 1, too unwell 1, declined 11		
*Cognition tested at both 1-3 months and 1 year n = 135*			
Age at stroke (IQR)	65 (56–72.5)		
Female gender	52 (39%)		
Stroke during follow-up	11 (8%)		

* Excludes a patient who started cognitive testing but only provided data on two ACE-R subtests.
