# Supplementary material for: Association between Striatal Brain Iron Deposition, Microbleeds and Cognition 1 Year After a Minor Ischaemic Stroke
Source: Int J Mol Sci. 2019 Mar 14;20(6):1293. doi: 10.3390/ijms20061293 (PMC6470500; doi:10.3390/ijms20061293)
Supplement: Supplementary file 1 [file ijms-20-01293-s001.pdf]

Association between Striatal Brain Iron Deposition and Microbleeds with Cognition 1 year after a Minor Ischaemic Stroke

Authors:

Maria del C. Valdés Hernández, Tessa Case, Francesca Chappell, Andreas Glatz, Stephen Makin, Fergus Doubal, and Joanna Wardlaw

SUPPLEMENTARY ANALYSES

All cognitive and imaging follow-up data (i.e. approximately one year after the stroke) were examined as follow: 1) with respect to the baseline data for missing (and not missing) values using IBM SPSS statistics Ver 21 (Release 21.0.0.0), 2) histograms and data distribution using MATLAB R2017b. To determine which probability distribution function fitted best each follow-up variable, the following distributions were evaluated for each case: Beta, Birnbaum-Saunders, Exponential, Extreme value, Gamma, Generalised extreme value, Generalized Pareto, Inverse Gaussian, Logistic, Log-logistic, Lognormal, Nakagami, Normal, Rayleigh, Rician, t location-scale, Weibull, Binomial, Negative binomial, and Poisson. Then, the results were ordered by: 1st) BIC - Bayesian information criterion, 2nd) NLogL - Negative of the log likelihood, 3rd) AIC - Akaike information criterion, and 4th) AICc - AIC with a correction for finite sample sizes. The best four fits were plotted.

The independence between covariates and the independent variable in the ANCOVA models was also evaluated using MATLABR2017b, as well as the possible interaction between the independent variable and the covariates, using Belsley collinearity diagnostics.

As result of the analyses described above:

1) ACE-R attention and orientation, ACE-R visuospatial abilities, and ACE-R language were transformed and, instead of ANCOVA models, multinomial logistic regression models were used to explore the influence of mineral deposition in these cognitive domains 1 year after stroke.
2) As expected, all cognitive variables were collinear among themselves, but were independent and did not interact with any covariate.

Baseline Sample Stroke subtype, arterial territory and cerebral hemisphere affected

Supplementary Table S2.1. Frequency of the index and old stroke lesion clusters per subtype (i.e. cortical vs. lacunar), arterial territory and cerebral hemisphere affected per patient at recruitment.

	Relevant stroke lesion– all ischaemic (see inclusion criteria)<br><br>Number of patients (n (%)) with index stroke lesion clusters of each subtype and in each location							
Number of lesion clusters in the sample (per patient)	Cortical lesion, MCA territory	Cortical lesion, ACA territory	Cortical lesion, PCA territory	Cortical lesion, border zones	Cortical lesion, cerebellum/ brain stem	Lacunar lesion	Right hemis-phere	Left hemis-phere
1	50 (18.9)	6 (2.3)	31 (11.7)	17 (6.4)	14 (5.3)	76 (28.8)	84 (31.8)	80 (30.3)
2	4 (1.5)		1 (0.4)	10 (3.8)	2 (0.8)	7 (2.7)	21 (8.0)	10 (3.8)
3							2 (0.8)	2 (0.8)
4								1 (0.4)
**Total number of patients**	**54 (20.5)**	**6 (2.3)**	**32 (12.1)**	**27 (10.2)**	**16 (6.1)**	**83 (31.4)**	**107 (40.5)**	**93 (35.2)**
Number of lesion clusters in the sample (per patient)	Old stroke lesion– 5 haemorrhagic and the rest ischaemic<br><br>Number of patients (n (%)) with old stroke lesion clusters of each subtype and in each location							
1	22 (8.3)		13 (4.9)	7 (2.7)	29 (11.0)	38 (14.4)	57 (21.6)	59 (22.3)
2	3 (1.1)	1 (0.4)	3 (1.1)	1 (0.4)	9 (3.4)	15 (5.7)	17 (6.4)	15 (5.7)
3	3 (1.1)			1 (0.4)	1 (0.4)	8 (3.0)	11 (4.2)	7 (2.7)
4						1 (0.4)	2 (0.8)	2 (0.8)
5						5 (1.9)		1 (0.4)
6								
7						2 (0.8)		
**Total number of patients**	**28 (10.60)**	**1 (0.4)**	**16 (6.06)**	**9 (3.4)**	**39 (14.8)**	**69 (26.1)**	**87 (33.0)**	**84 (31.8)**

Note: This table refers to the ischaemic and haemorrhagic lesion clusters due to the index and old strokes identified (and computationally quantified) in the images, not to the stroke events.

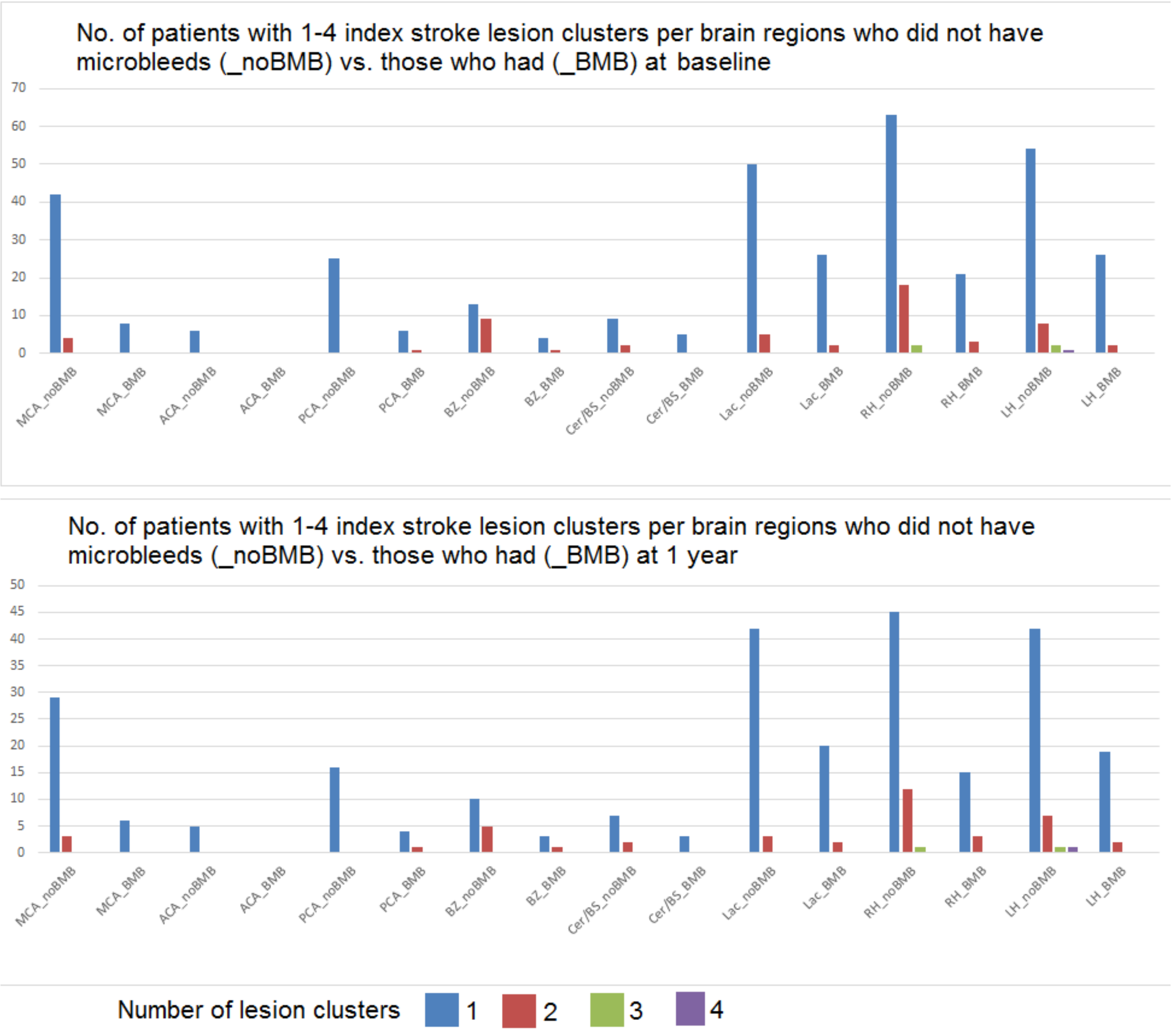

Figure S2.1. Bar Graphs of each of the index stroke lesion clusters per hemisphere/site in the presence or absence of microbleeds (i.e. BMB vs. No BMB) at baseline and 1 year after the index stroke. MCA_noBMB: no. of patients that did not have microbleeds with 1-4 index stroke cortical lesion clusters in the Middle Cerebral Artery (MCA) territory; MCA_BMB: no. of patients that had at least 1 microbleed with 1-4 index stroke cortical lesion clusters in the MCA territory; ACA_noBMB: no. of patients that did not have microbleeds with 1-4 index stroke cortical lesion clusters in the Anterior Cerebral Artery (ACA) territory; ACA_BMB: no. of patients that had at least 1 microbleed with 1-4 index stroke cortical lesion clusters in the ACA territory. Following the same format: PCA_noBMB and PCA_BMB refers to similar information but for the Posterior Cerebral Artery territory, BZ_noBMB and BZ_BMB refers to similar information for the Border Zones (i.e. watershed regions), Cer/BS_noBMB and Cer/BS_BMB refers to similar information for the cerebellum and brain stem, Lac_noBMB and Lac_BMB refers to similar information but for lacunar stroke lesions in the subcortical regions, optical radiations and pons, RH and LH groups similar information in the Right and Left Hemispheres.

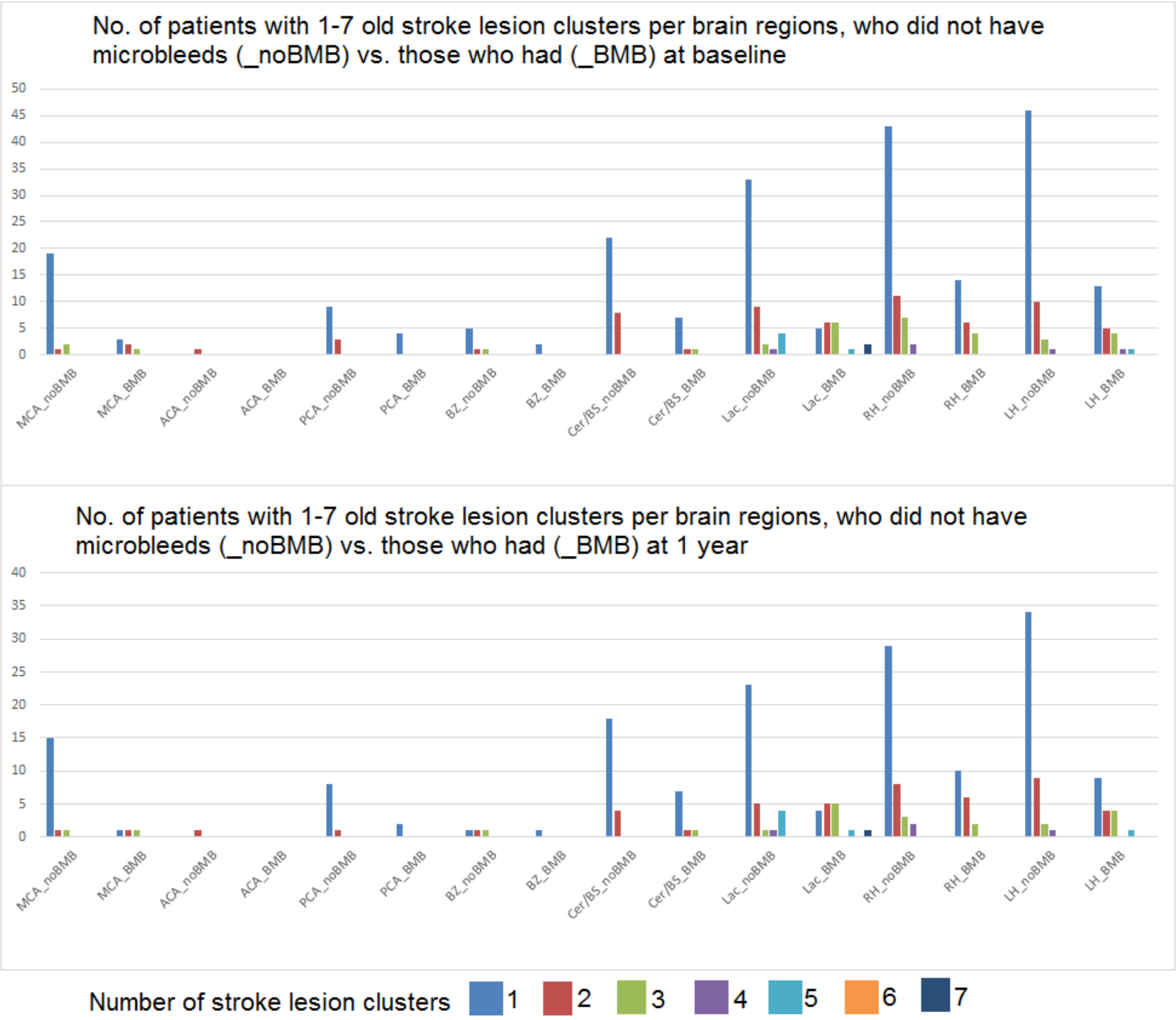

Figure S2.2. Bar Graphs of each of the old stroke lesion clusters per hemisphere/site in the presence or absence of microbleeds (i.e. BMB vs. No BMB) at baseline and 1 year after the index stroke. MCA_noBMB: no. of patients that did not have microbleeds with 1-7 old stroke cortical lesion clusters in the Middle Cerebral Artery (MCA) territory; MCA_BMB: no. of patients that had at least 1 microbleed with 1-7 old stroke cortical lesion clusters in the MCA territory; ACA_noBMB: no. of patients that did not have microbleeds with 1-7 old stroke cortical lesion clusters in the Anterior Cerebral Artery (ACA) territory; ACA_BMB: no. of patients that had at least 1 microbleed with 1-7 old stroke cortical lesion clusters in the ACA territory. Following the same format: PCA_noBMB and PCA_BMB refers to similar information but for the Posterior Cerebral Artery territory, BZ_noBMB and BZ_BMB refers to similar information for the Border Zones (i.e. watershed regions), Cer/BS_noBMB and Cer/BS_BMB refers to similar information for the cerebellum and brain stem, Lac_noBMB and Lac_BMB refers to similar information but for lacunar stroke lesions in the subcortical regions, optical radiations and pons, RH and LH groups similar information in the Right and Left Hemispheres.

Cognitive variables

ACE-R 1-year follow-up (ACER_2tp) examined with respect to the first wave of cognitive testing (ACER_1tp)

There are no valid cases for ACER_2tp when ACER_1tp = 59.000.
ACER_2tp is constant when ACER_1tp = 69.00.
ACER_2tp is constant when ACER_1tp = 70.00.
ACER_2tp is constant when ACER_1tp = 72.00.
ACER_2tp is constant when ACER_1tp = 85.00.
ACER_2tp is constant when ACER_1tp = 87.00.

Case Processing Summary

	ACER_1tp	Cases					
		Valid		Missing		Total	
		N	Percent	N	Percent	N	Percent
ACER_2tp	69.00	1	50.0%	1	50.0%	2	100.0%
	70.00	1	50.0%	1	50.0%	2	100.0%
	71.00	2	100.0%	0	0.0%	2	100.0%
	72.00	1	33.3%	2	66.7%	3	100.0%
	75.00	4	100.0%	0	0.0%	4	100.0%
	76.00	3	75.0%	1	25.0%	4	100.0%
	78.00	5	83.3%	1	16.7%	6	100.0%
	80.00	4	66.7%	2	33.3%	6	100.0%
	81.00	6	100.0%	0	0.0%	6	100.0%
	82.00	2	100.0%	0	0.0%	2	100.0%
	83.00	3	60.0%	2	40.0%	5	100.0%
	84.00	4	80.0%	1	20.0%	5	100.0%
	85.00	1	33.3%	2	66.7%	3	100.0%
	86.00	7	87.5%	1	12.5%	8	100.0%
	87.00	1	50.0%	1	50.0%	2	100.0%
	88.00	5	100.0%	0	0.0%	5	100.0%
	89.00	5	62.5%	3	37.5%	8	100.0%
	90.00	9	100.0%	0	0.0%	9	100.0%
	91.00	7	100.0%	0	0.0%	7	100.0%
	92.00	9	100.0%	0	0.0%	9	100.0%
	93.00	9	100.0%	0	0.0%	9	100.0%
	94.00	9	90.0%	1	10.0%	10	100.0%
	95.00	10	90.9%	1	9.1%	11	100.0%

96.00	10	100.0%	0	0.0%	10	100.0%
97.00	3	100.0%	0	0.0%	3	100.0%
98.00	7	100.0%	0	0.0%	7	100.0%
99.00	5	100.0%	0	0.0%	5	100.0%
100.00	2	66.7%	1	33.3%	3	100.0%

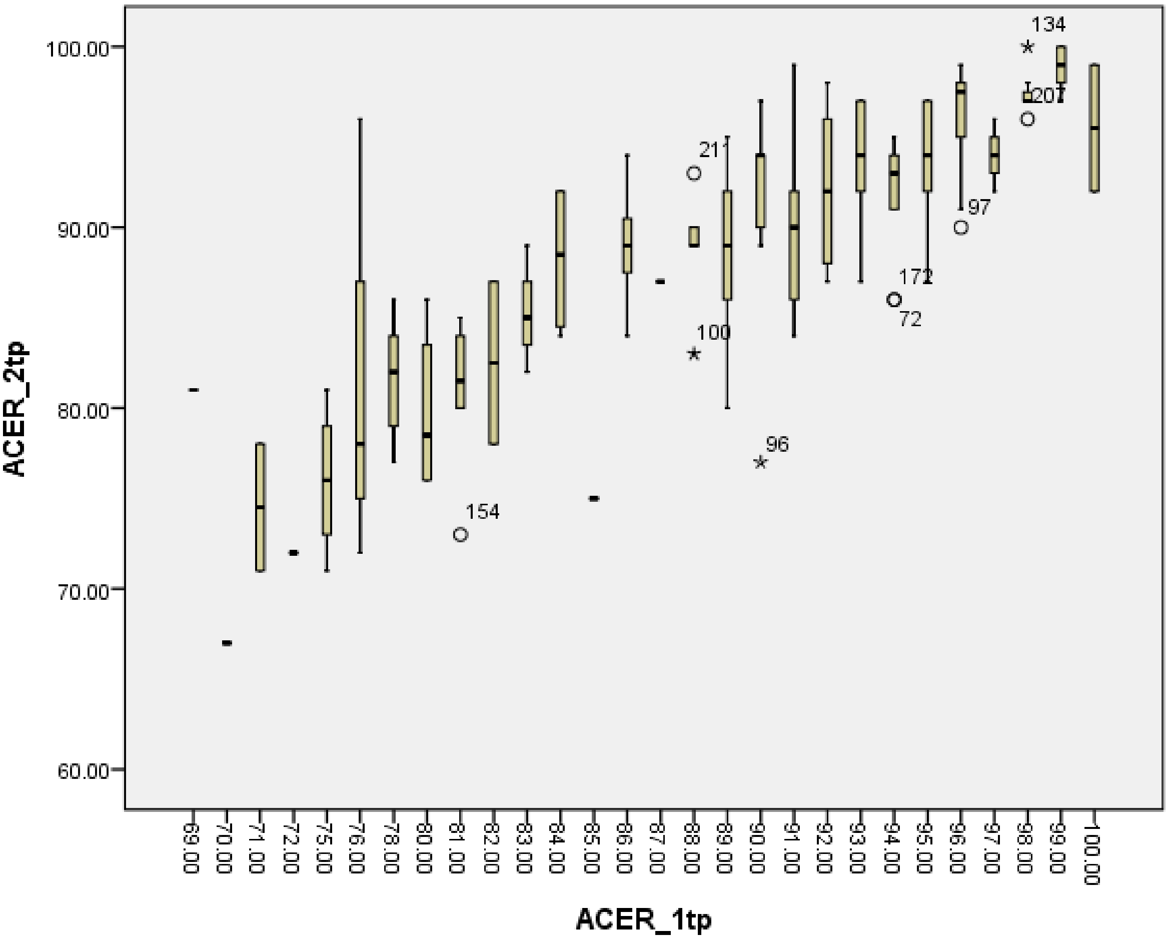

Figure S1.1. ACE-R follow-up with respect to ACE-R results at first wave of cognitive testing

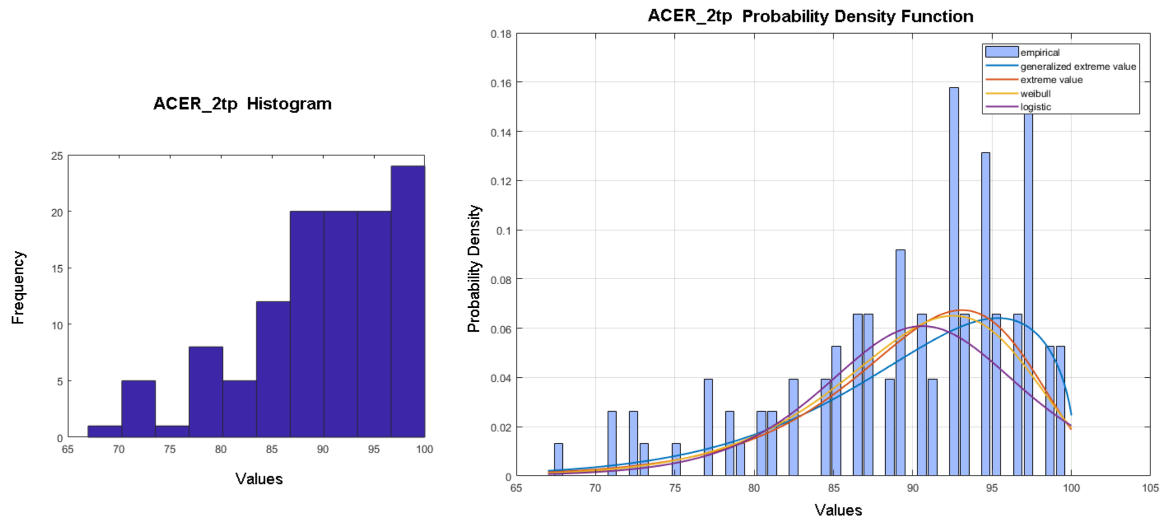

Figure S1.2. Histogram and matching curves of the four probability density functions that produced the best fit for ACE-R general follow-up: generalised extreme value distribution (NlogL=383.3, BIC=780.9, AIC=772.6), extreme value distribution (NLogL=388.2, BIC=785.9, AIC=780.4), Weibull distribution (NLogL=389.6, BIC=788.8, AIC=783.2), and logistic (NLogL=400.7, BIC=811.0, AIC=805.5).

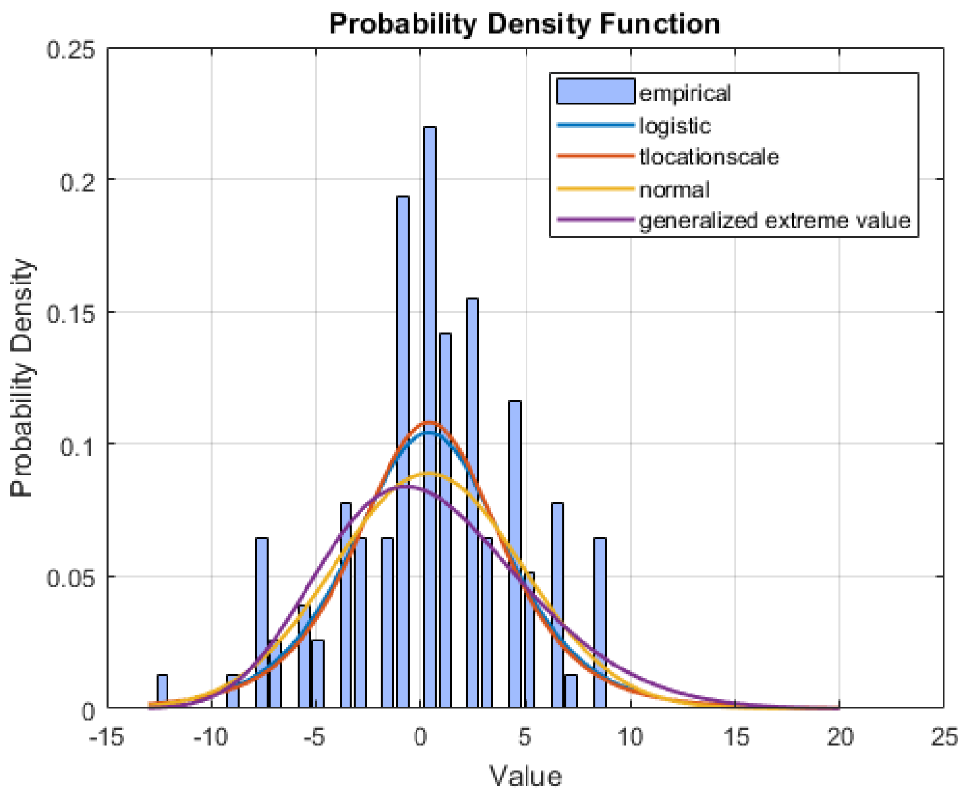

Figure S1.3. Matching curves of the four probability density functions that produced the best fit for ACE-R change (ACE-R change = ACER_2tp – ACER_1tp): logistic distribution (NLogL=337.4, BIC=684.4, AIC=678.9), tlocation scale distribution (NLogL=337.1, BIC=688.6, AIC=680.3), normal distribution (NLogL=341.2, BIC=692.0, AIC=686.5), and generalised extreme value distribution (NLogL=344.0, BIC=702.3, AIC=694.1).

ACE-R attention and orientation 1-year follow-up (Orientation_2tp) examined with respect to the first wave of cognitive testing (Orientation_1tp)

Orientation_2tp is constant when Orientation_1tp = 12.00.

Orientation_2tp is constant when Orientation_1tp = 14.00.

Case Processing Summary

	Orientation_1tp	Cases				
		Valid		Missing		Total
		N	Percent	N	Percent	N
Orientation_2tp	12.00	1	100.0%	0	0.0%	1
	13.00	2	100.0%	0	0.0%	2
	14.00	1	100.0%	0	0.0%	1
	15.00	6	85.7%	1	14.3%	7
	16.00	8	72.7%	3	27.3%	11
	17.00	29	80.6%	7	19.4%	36
	18.00	89	89.0%	11	11.0%	100

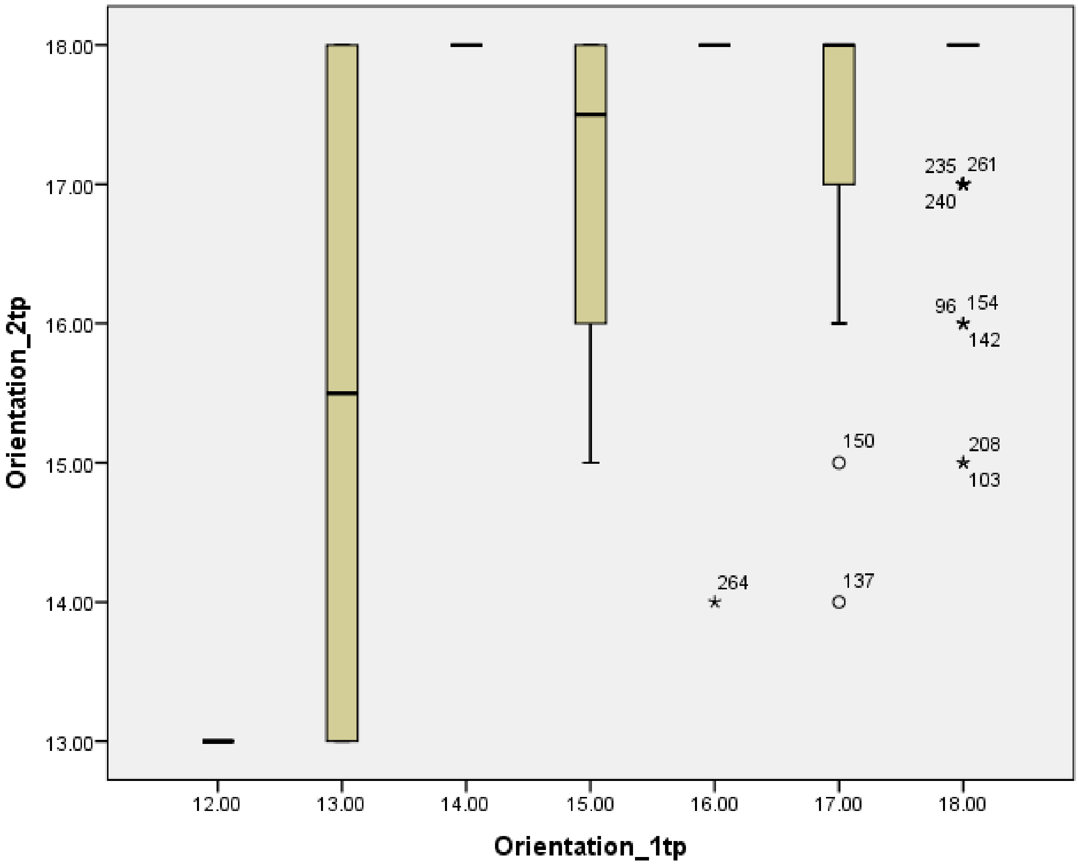

Figure S1.4. ACE-R attention and orientation test follow-up with respect to ACE-R attention and orientation test results at first wave of cognitive testing

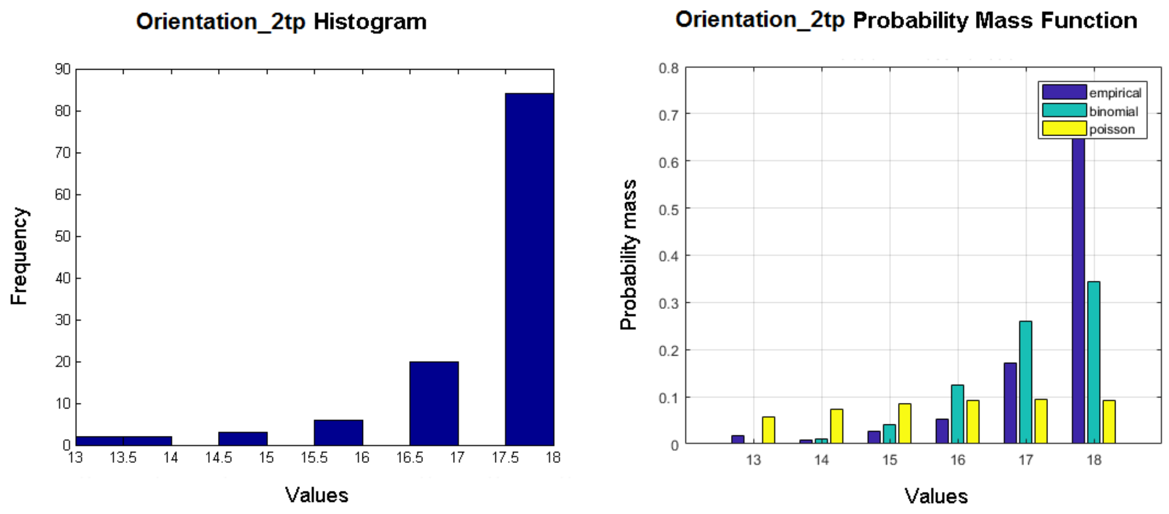

Figure S1.5. Histogram and matching histograms of the two probability mass functions that produced the best fit for ACE-R attention and orientation follow-up: binomial distribution (NLogL=159.8, BIC=329.2, AICc=323.8), and Poisson distribution (NLogL=279.1, BIC=563.0, AICc=560.2).

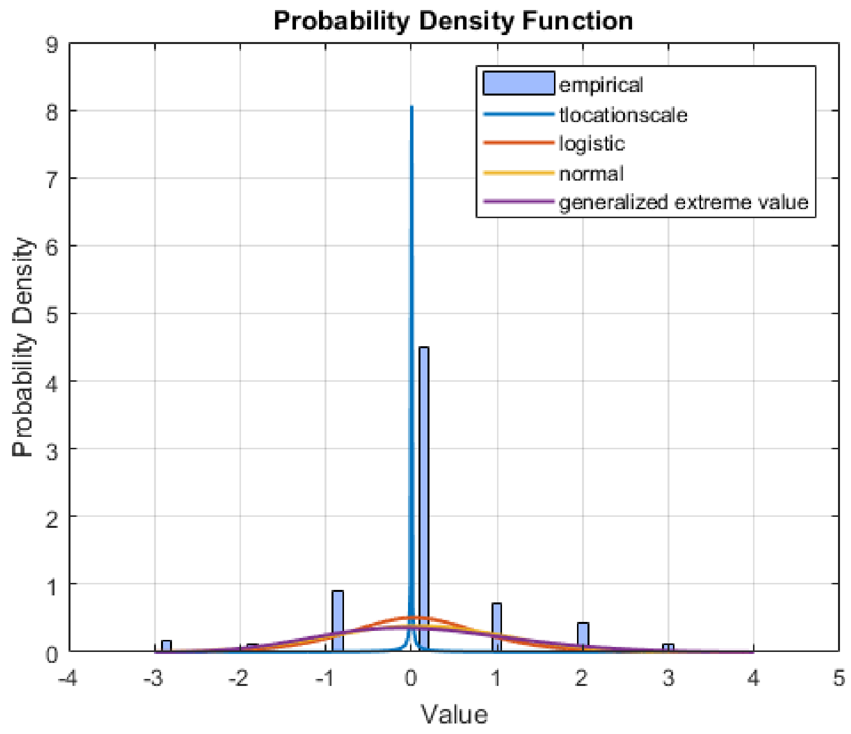

Figure S1.6. Matching curves of the four probability density functions that produced the best fit for ACE-R attention and orientation change (Orientation change = Orientation_2tp – Orientation_1tp): logistic distribution (NLogL=160.7, BIC=330.9, AICc=325.5), tlocation scale distribution (NLogL=-504.3, BIC=-994.2, AICc=-1002.3), normal distribution (NLogL=171.1, BIC=351.8, AICc=346.4), and generalised extreme value distribution (NLogL=174.2, BIC=362.7, AICc=354.7).

ACE-R memory 1-year follow-up (Memory_2tp) examined with respect to the first wave of cognitive testing (Memory_1tp)

There are no valid cases for Memory_2tp when Memory_1tp = 8.000.

Memory_2tp is constant when Memory_1tp = 9.00.

Case Processing Summary

	Memory_1tp	Cases					
		Valid		Missing		Total	
		N	Percent	N	Percent	N	Percent
Memory_2tp	9.00	1	50.0%	1	50.0%	2	100.0%
	11.00	4	100.0%	0	0.0%	4	100.0%
	14.00	3	100.0%	0	0.0%	3	100.0%
	15.00	4	80.0%	1	20.0%	5	100.0%
	16.00	10	100.0%	0	0.0%	10	100.0%
	17.00	6	100.0%	0	0.0%	6	100.0%
	18.00	8	72.7%	3	27.3%	11	100.0%
	19.00	6	85.7%	1	14.3%	7	100.0%
	20.00	6	75.0%	2	25.0%	8	100.0%
	21.00	9	90.0%	1	10.0%	10	100.0%
	22.00	11	84.6%	2	15.4%	13	100.0%
	23.00	10	90.9%	1	9.1%	11	100.0%
	24.00	23	92.0%	2	8.0%	25	100.0%
	25.00	18	94.7%	1	5.3%	19	100.0%
	26.00	16	94.1%	1	5.9%	17	100.0%

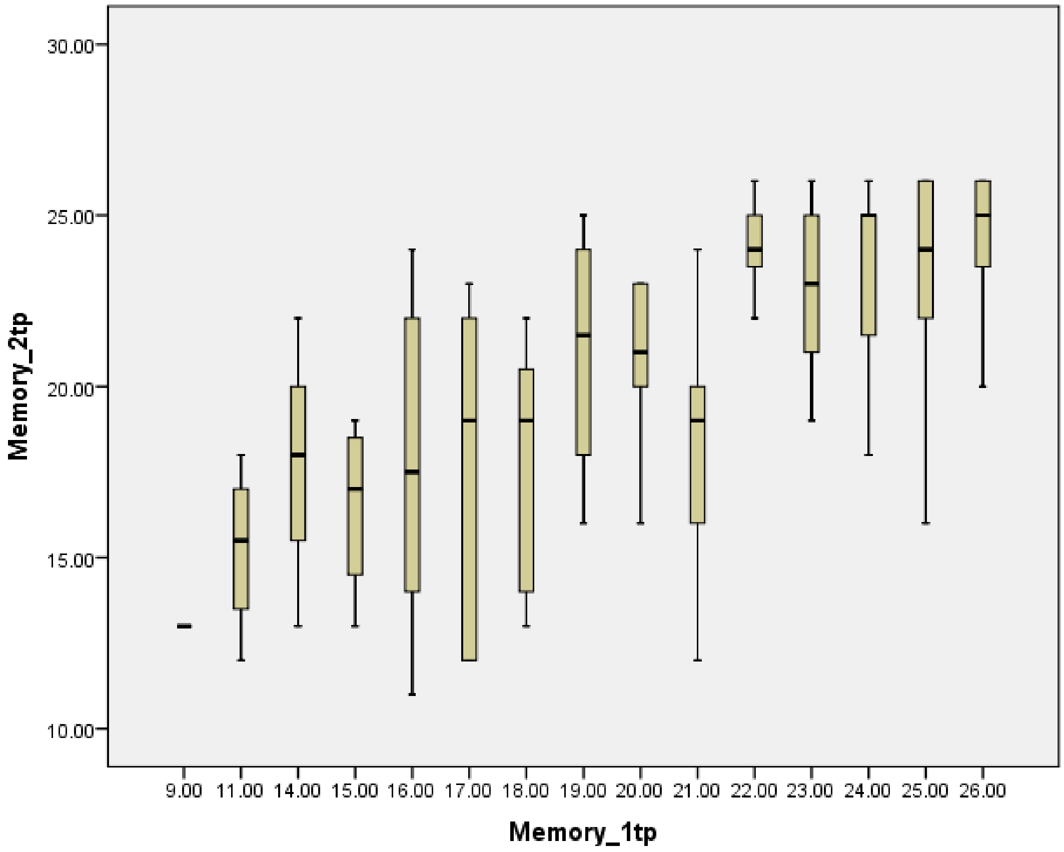

Figure S1.7. ACE-R memory test follow-up with respect to ACE-R memory test results at first wave of cognitive testing

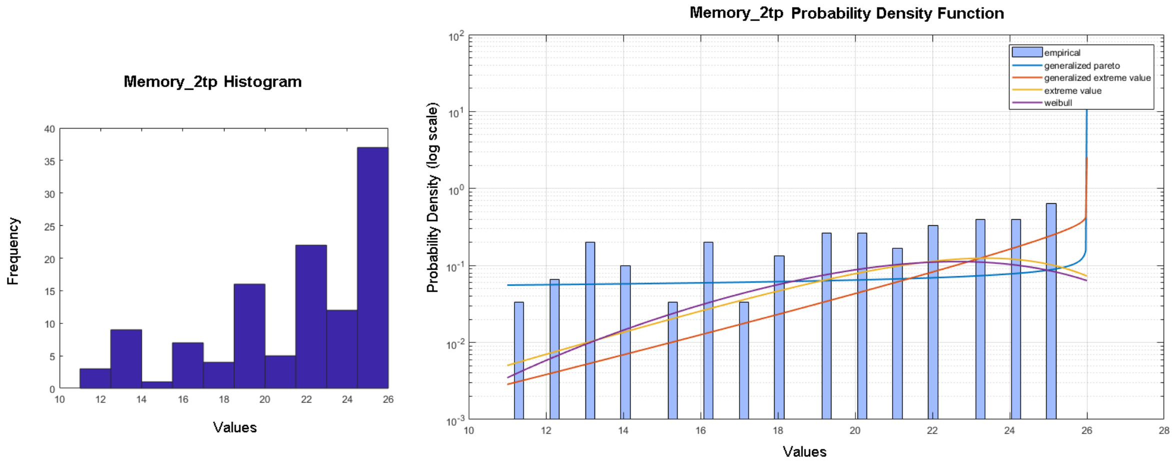

Figure S1.8. Histogram and matching curves of the four probability density functions that produced the best fit for ACE-R memory follow-up: generalised extreme value distribution (NLogL=219.3, BIC=452.8, AIC=444.6), extreme value distribution (NLogL=316.8, BIC=643.1, AIC=637.6), Weibull distribution (NLogL=322.2, BIC=653.8, AIC=648.3), and generalised Pareto distribution (NLogL=220.3, BIC=454.9, AIC=446.6).

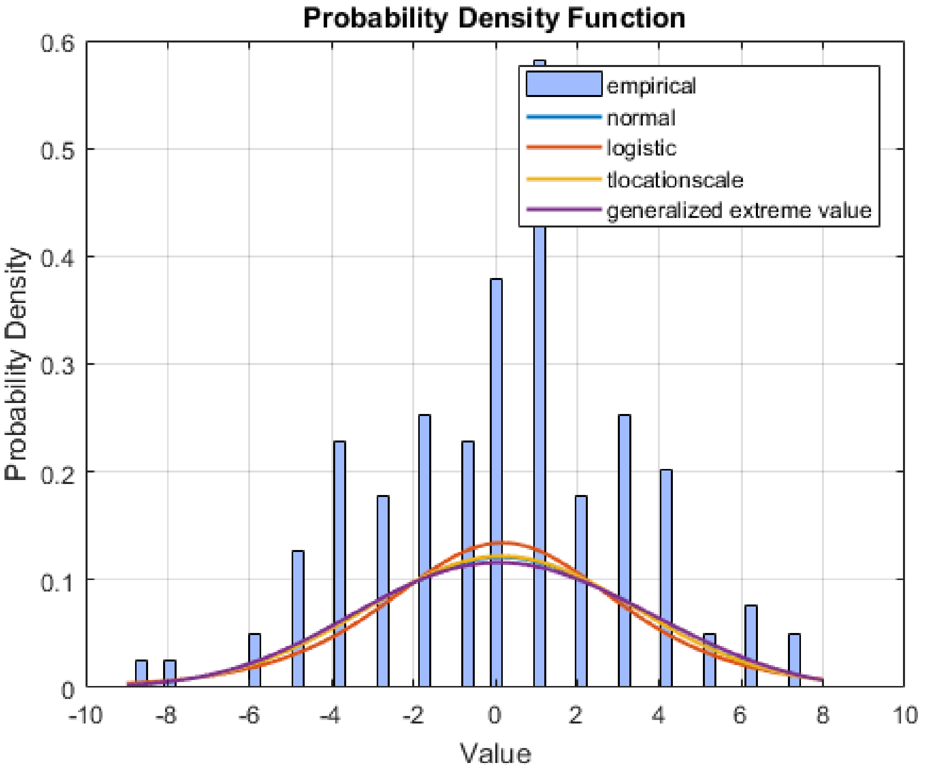

Figure S1.9. Matching curves of the four probability density functions that produced the best fit for ACE-R memory change (Memory change = Memory_2tp – Memory_1tp): normal distribution (NLogL=304.5, BIC=618.6, AIC=613.0), tlocation scale distribution (NLogL=304.5, BIC=623.3, AIC=615.0), logistic distribution (NLogL=305.1, BIC=619.8, AIC=614.3), and generalised extreme value distribution (NLogL=304.6, BIC=623.6, AIC=615.3).

ACE-R verbal fluency 1-year follow-up (Fluency_2tp) examined with respect to the first wave of cognitive testing (Fluency_1tp)

There are no valid cases for Fluency_2tp when Fluency_1tp = 2.000.

Fluency_2tp is constant when Fluency_1tp = 4.00.

Case Processing Summary

	Fluency_1tp	Cases					
		Valid		Missing		Total	
		N	Percent	N	Percent	N	Percent
Fluency_2tp	4.00	1	100.0%	0	0.0%	1	100.0%
	5.00	6	100.0%	0	0.0%	6	100.0%
	6.00	8	66.7%	4	33.3%	12	100.0%
	7.00	5	71.4%	2	28.6%	7	100.0%
	8.00	8	80.0%	2	20.0%	10	100.0%

	9.00	12	80.0%	3	20.0%	15	100.0%
	10.00	19	90.5%	2	9.5%	21	100.0%
	11.00	21	84.0%	4	16.0%	25	100.0%
	12.00	16	88.9%	2	11.1%	18	100.0%
	13.00	23	100.0%	0	0.0%	23	100.0%
	14.00	16	88.9%	2	11.1%	18	100.0%

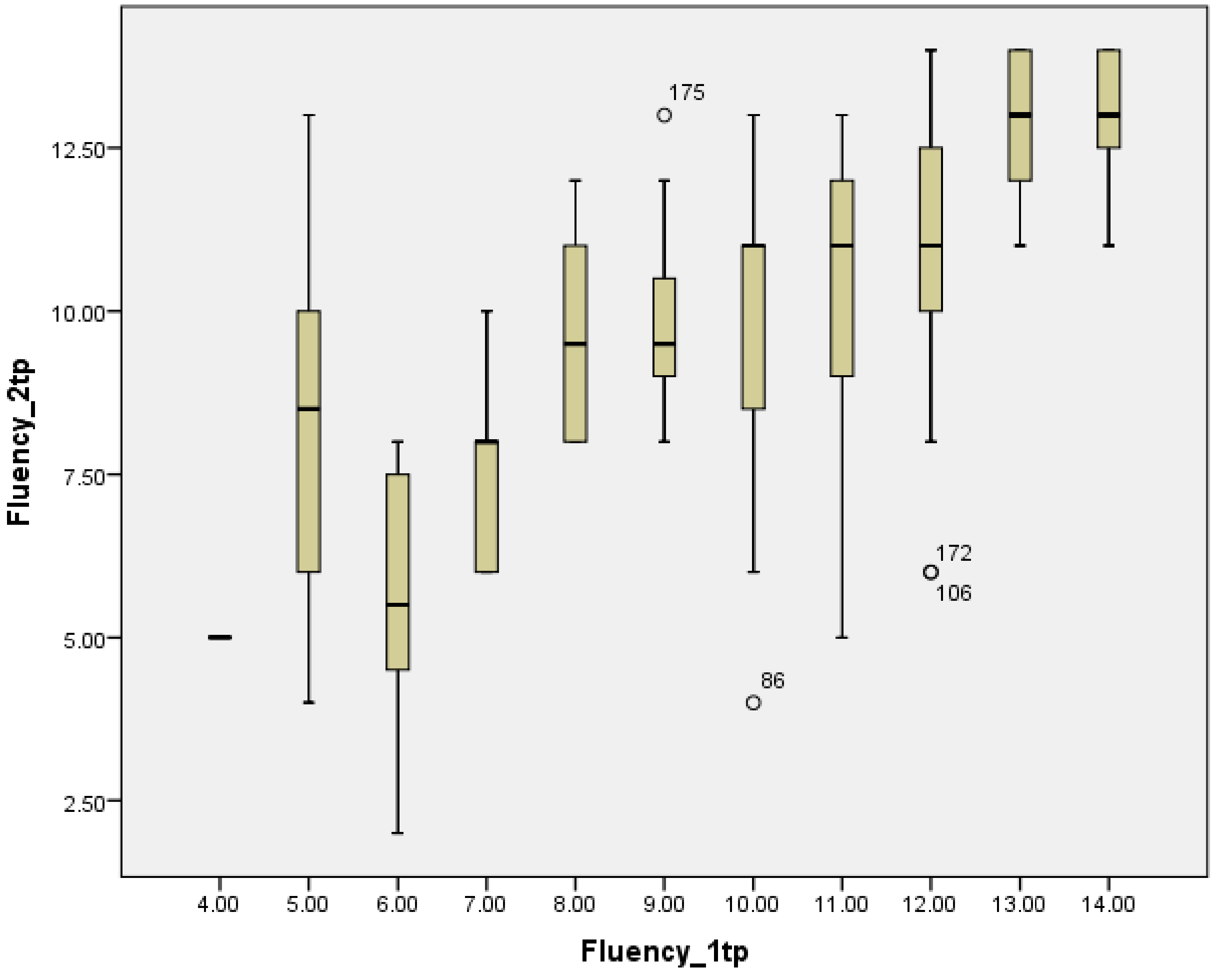

Figure S1.10. ACE-R verbal fluency test follow-up with respect to ACE-R verbal fluency test results at first wave of cognitive testing

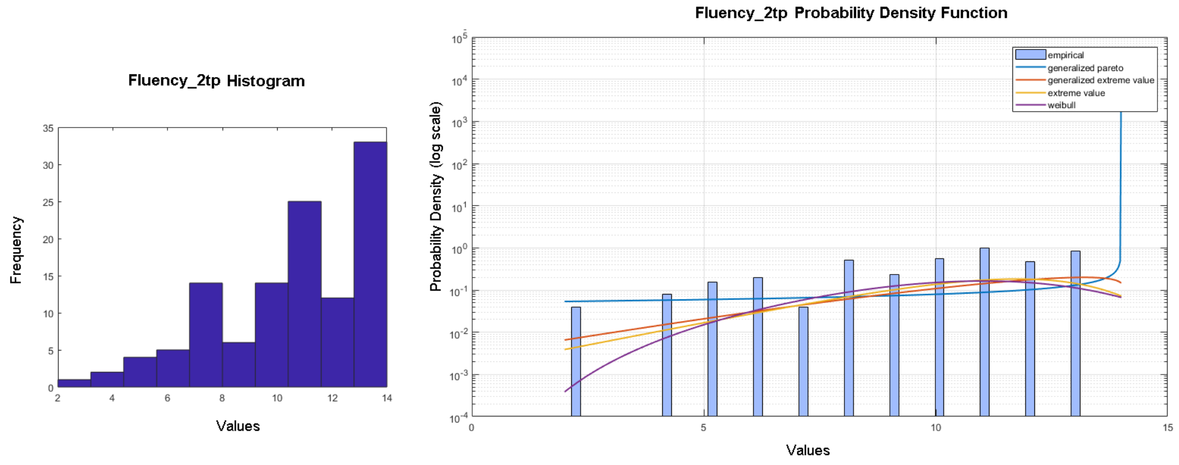

Figure S1.11. Histogram and matching curves of the four probability density functions that produced the best fit for ACE-R verbal fluency follow-up: generalised extreme value distribution (NLogL=263.9, BIC=542.0, AIC=533.7), extreme value distribution (NLogL=270.8, BIC=551.2, AIC=545.6), Weibull distribution (NLogL=277.5, BIC=564.6, AIC=559.1), and generalised Pareto distribution (NLogL=175.9, BIC=366.2, AIC=357.9).

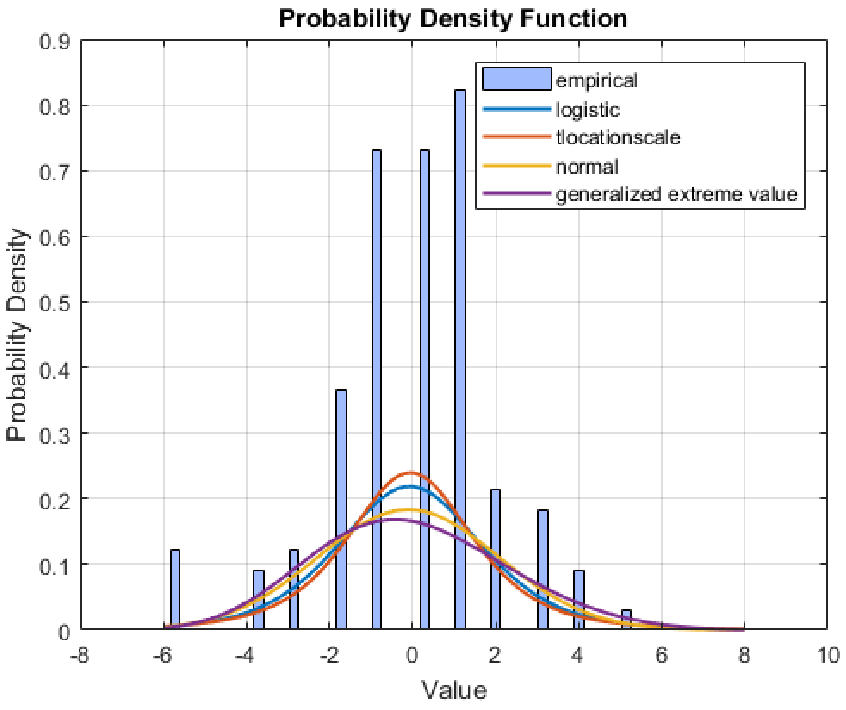

Figure S1.12. Matching curves of the four probability density functions that produced the best fit for ACE-R verbal fluency change (Fluency change = Fluency_2tp – Fluency_1tp): normal distribution (NLogL=256.4, BIC=522.3, AIC=516.8), tlocation scale distribution (NLogL=2501.0, BIC=516.3, AIC=508.0), logistic distribution (NLogL=251.8, BIC=513.1, AIC=507.6), and generalised extreme value distribution (NLogL=260.3, BIC=534.9, AIC=526.6).

ACE-R language 1-year follow-up (Language_2tp) examined with respect to the first wave of cognitive testing (Language_1tp)

Language_2tp is constant when Language_1tp = 19.00.

Case Processing Summary

	Language_1tp	Cases					
		Valid		Missing		Total	
		N	Percent	N	Percent	N	Percent
Language_2tp	19.00	1	50.0%	1	50.0%	2	100.0%
	21.00	2	50.0%	2	50.0%	4	100.0%
	22.00	5	62.5%	3	37.5%	8	100.0%
	23.00	17	89.5%	2	10.5%	19	100.0%
	24.00	23	82.1%	5	17.9%	28	100.0%
	25.00	27	87.1%	4	12.9%	31	100.0%
	26.00	60	92.3%	5	7.7%	65	100.0%

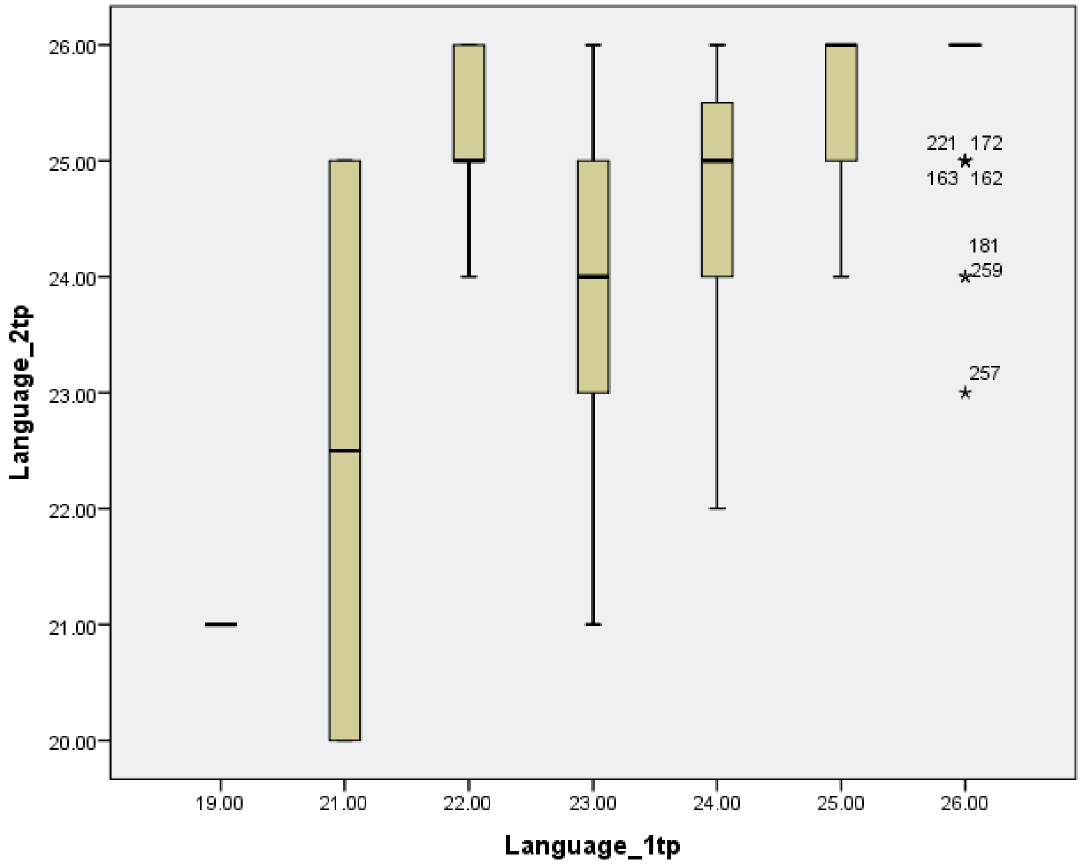

Figure S1.13. ACE-R language test follow-up with respect to ACE-R language test results at first wave of cognitive testing

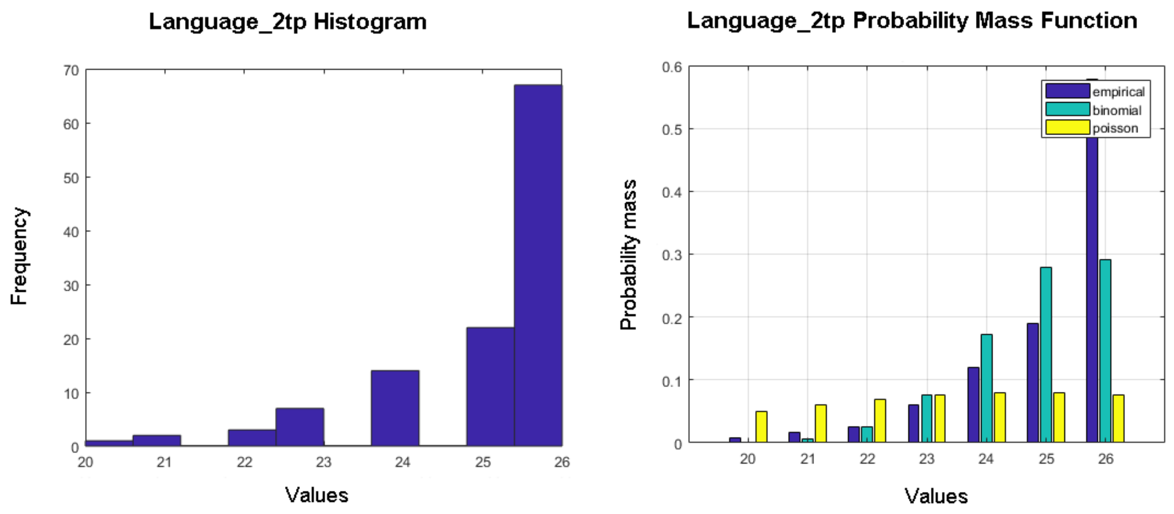

Figure S1.14. Histogram and matching curves of the two probability mass (i.e. discrete) functions that produced the best fit for ACE-R language follow-up: binomial distribution (NLogL=182.4, BIC=374.4, AICc=369.0) and Poisson distribution (NLogL=300.4, BIC=605.5, AICc=602.8).

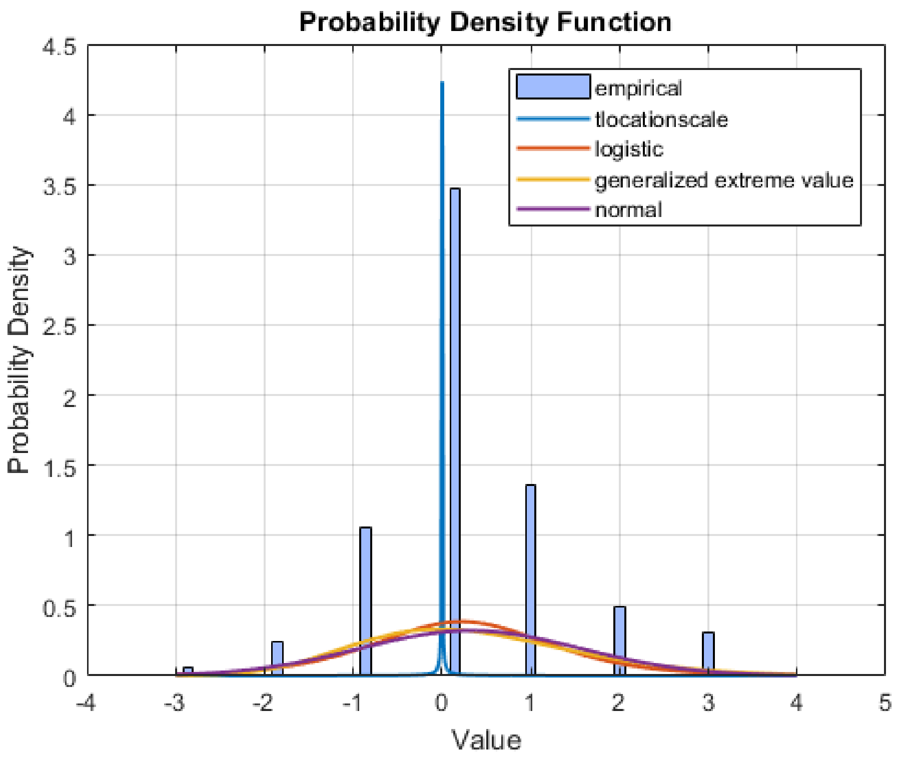

Figure S1.15. Matching curves of the four probability density functions that produced the best fit for ACE-R language change (Language change = Language_2tp – Language_1tp): normal distribution (NLogL=190.9, BIC=391.3, AIC=385.7), tlocation scale distribution (NLogL=-288.9, BIC=-563.5, AIC=-571.7), logistic distribution (NLogL=186.5, BIC=382.5, AIC=377.0), and generalised extreme value distribution (NLogL=187.5, BIC=389.2, AIC=380.9).

ACE-R visuospatial ability 1-year follow-up (Visuospatial_2tp) examined with respect to the first wave of cognitive testing (Visuospatial_1tp)

Visuospatial_2tp is constant when Visuospatial_1tp = 8.00.

There are no valid cases for Visuospatial_2tp when Visuospatial_1tp = 11.000.

Case Processing Summary

	Visuospatial_1tp	Cases				
		Valid		Missing		Total
		N	Percent	N	Percent	N
Visuospatial_2tp	8.00	1	50.0%	1	50.0%	2
	10.00	4	100.0%	0	0.0%	4
	12.00	3	50.0%	3	50.0%	6
	13.00	5	83.3%	1	16.7%	6
	14.00	19	76.0%	6	24.0%	25
	15.00	37	86.0%	6	14.0%	43
	16.00	66	94.3%	4	5.7%	70

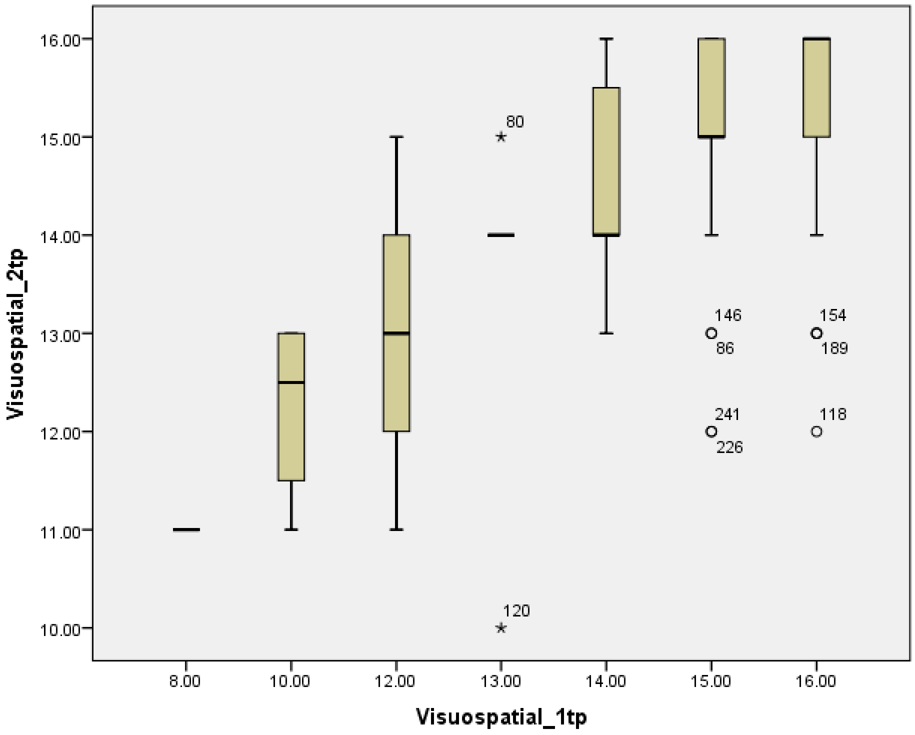

Figure S1.16. ACE-R visuospatial ability test follow-up with respect to ACE-R visuospatial ability test results at first wave of cognitive testing

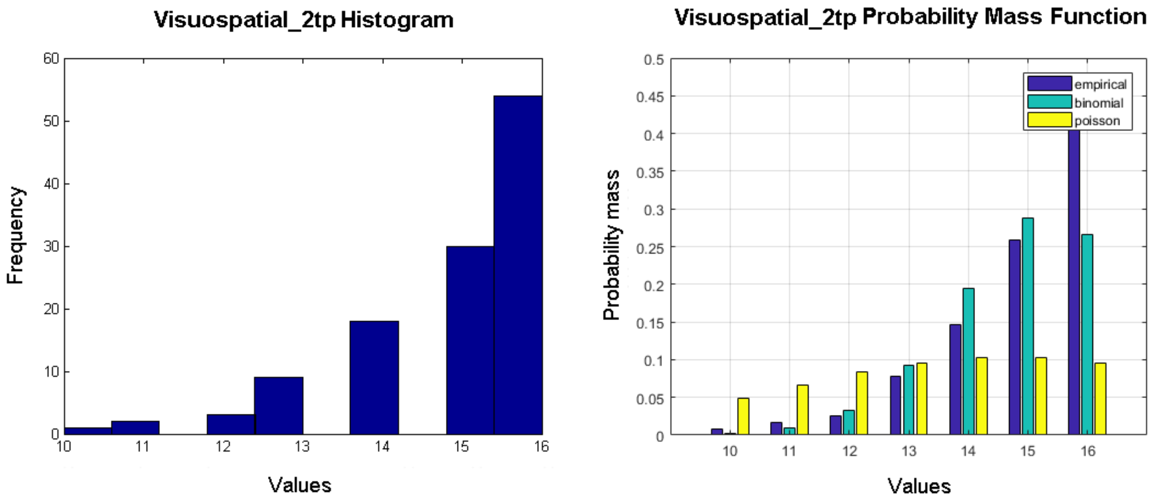

Figure S1.17. Histogram (left) and matching curves of the two probability mass (i.e. discrete) functions (right) that produced the best fit for ACE-R visuospatial abilities follow-up: binomial distribution (NLogL=185.8, BIC=381.1, AICc=375.7) and Poisson distribution (NLogL=272.9, BIC=550.6, AICc=547.9).

NART 1-year follow-up (NART_2tp) examined with respect to the first wave of cognitive testing (NART_1tp)

There are no valid cases for NART_2tp when NART_1tp = 7.000.

NART_2tp is constant when NART_1tp = 10.00.

NART_2tp is constant when NART_1tp = 15.00.

NART_2tp is constant when NART_1tp = 20.00.

NART_2tp is constant when NART_1tp = 23.00.

NART_2tp is constant when NART_1tp = 28.00.

NART_2tp is constant when NART_1tp = 34.00.

NART_2tp is constant when NART_1tp = 49.00.

Case Processing Summary

	NART_1tp	Cases					
		Valid		Missing		Total	
		N	Percent	N	Percent	N	Percent
NART_2tp	10.00	1	100.0%	0	0.0%	1	100.0%
	13.00	2	100.0%	0	0.0%	2	100.0%
	14.00	3	100.0%	0	0.0%	3	100.0%

	15.00	1	50.0%	1	50.0%	2	100.0%
	16.00	2	100.0%	0	0.0%	2	100.0%
	17.00	2	100.0%	0	0.0%	2	100.0%
	20.00	1	50.0%	1	50.0%	2	100.0%
	22.00	2	100.0%	0	0.0%	2	100.0%
	23.00	1	50.0%	1	50.0%	2	100.0%
	24.00	2	100.0%	0	0.0%	2	100.0%
	25.00	2	50.0%	2	50.0%	4	100.0%
	26.00	2	100.0%	0	0.0%	2	100.0%
	27.00	2	50.0%	2	50.0%	4	100.0%
	28.00	1	50.0%	1	50.0%	2	100.0%
	29.00	4	80.0%	1	20.0%	5	100.0%
	30.00	4	80.0%	1	20.0%	5	100.0%
	31.00	4	100.0%	0	0.0%	4	100.0%
	32.00	3	75.0%	1	25.0%	4	100.0%
	33.00	3	60.0%	2	40.0%	5	100.0%
	34.00	1	33.3%	2	66.7%	3	100.0%
	35.00	5	83.3%	1	16.7%	6	100.0%
	36.00	4	100.0%	0	0.0%	4	100.0%
	37.00	4	100.0%	0	0.0%	4	100.0%
	38.00	6	100.0%	0	0.0%	6	100.0%
	39.00	5	100.0%	0	0.0%	5	100.0%
	40.00	4	66.7%	2	33.3%	6	100.0%
	41.00	6	85.7%	1	14.3%	7	100.0%
	42.00	8	88.9%	1	11.1%	9	100.0%
	43.00	5	83.3%	1	16.7%	6	100.0%
	44.00	6	85.7%	1	14.3%	7	100.0%
	45.00	5	83.3%	1	16.7%	6	100.0%
	46.00	5	83.3%	1	16.7%	6	100.0%
	47.00	8	88.9%	1	11.1%	9	100.0%
	48.00	5	100.0%	0	0.0%	5	100.0%
	49.00	1	100.0%	0	0.0%	1	100.0%
	50.00	4	100.0%	0	0.0%	4	100.0%

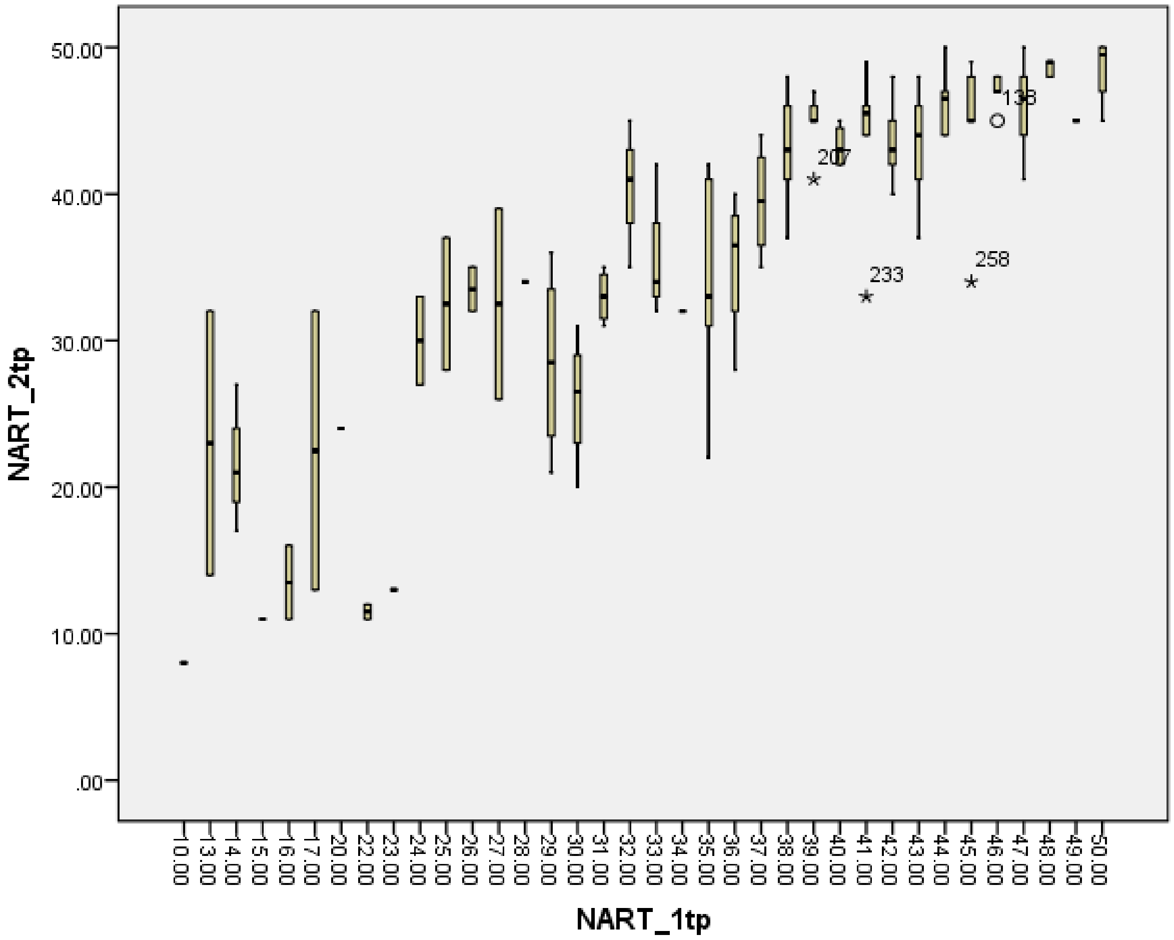

Figure S1.18. NART follow-up with respect to NART results at first wave of cognitive testing

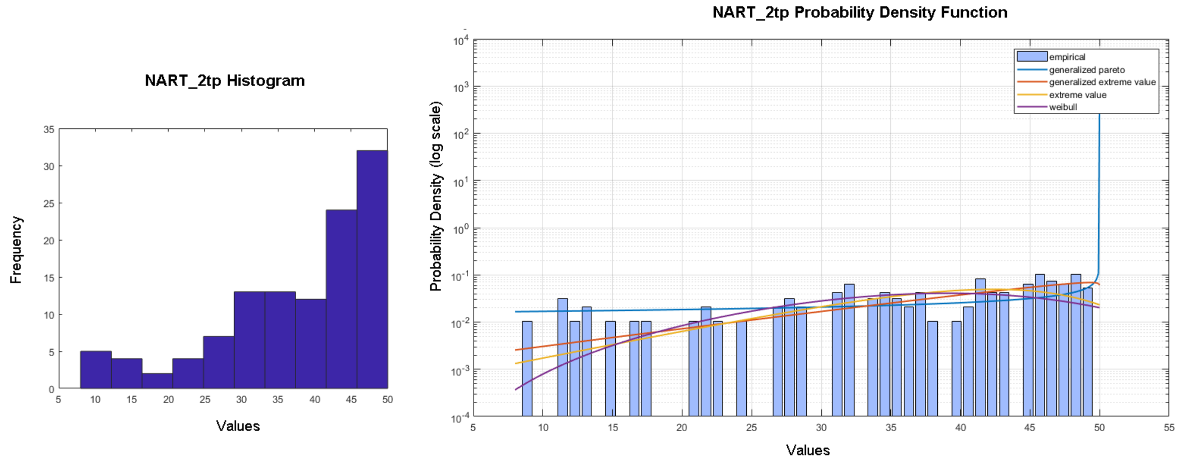

Figure S1.19. Histogram and matching curves of the four probability density functions that produced the best fit for NART follow-up: generalised extreme value distribution (NLogL=412.0, BIC=838.4, AIC=830.1), extreme value distribution (NLogL=427.0, BIC=863.5, AIC=857.9), Weibull distribution (NLogL=440.2, BIC=889.9, AIC=884.4), and generalised Pareto distribution (NLogL=374.5, BIC=763.3, AIC=755.0).

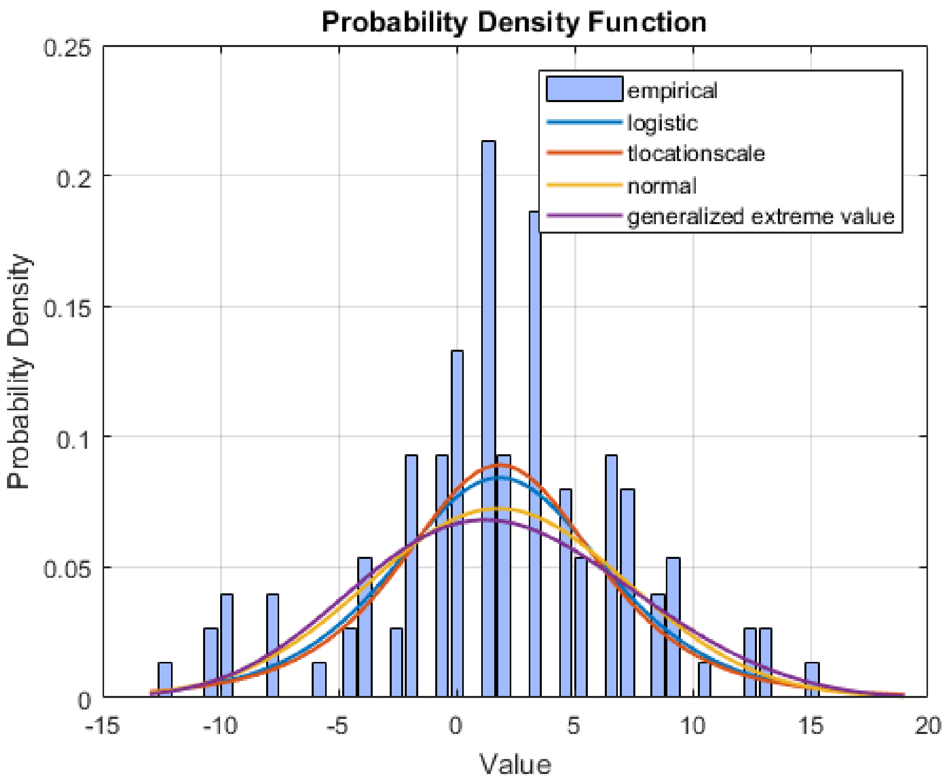

Figure S1.20. Matching curves of the four probability density functions that produced the best fit for NART change (NART change = NART_2tp – NART_1tp): normal distribution (NLogL=365.0, BIC=739.6, AIC=734.1), tlocation scale distribution (NLogL=362.8, BIC=740.0, AIC=731.7), logistic distribution (NLogL=362.7, BIC=735.0, AIC=729.5), and generalised extreme value distribution (NLogL=366.9, BIC=748.2, AIC=739.9).

Imaging variables

Volume of brain microbleeds and other haemorrhages at 1-year follow-up (BMBinICV_2tp) examined with respect to the baseline measurements (BMBinICV_1tp)

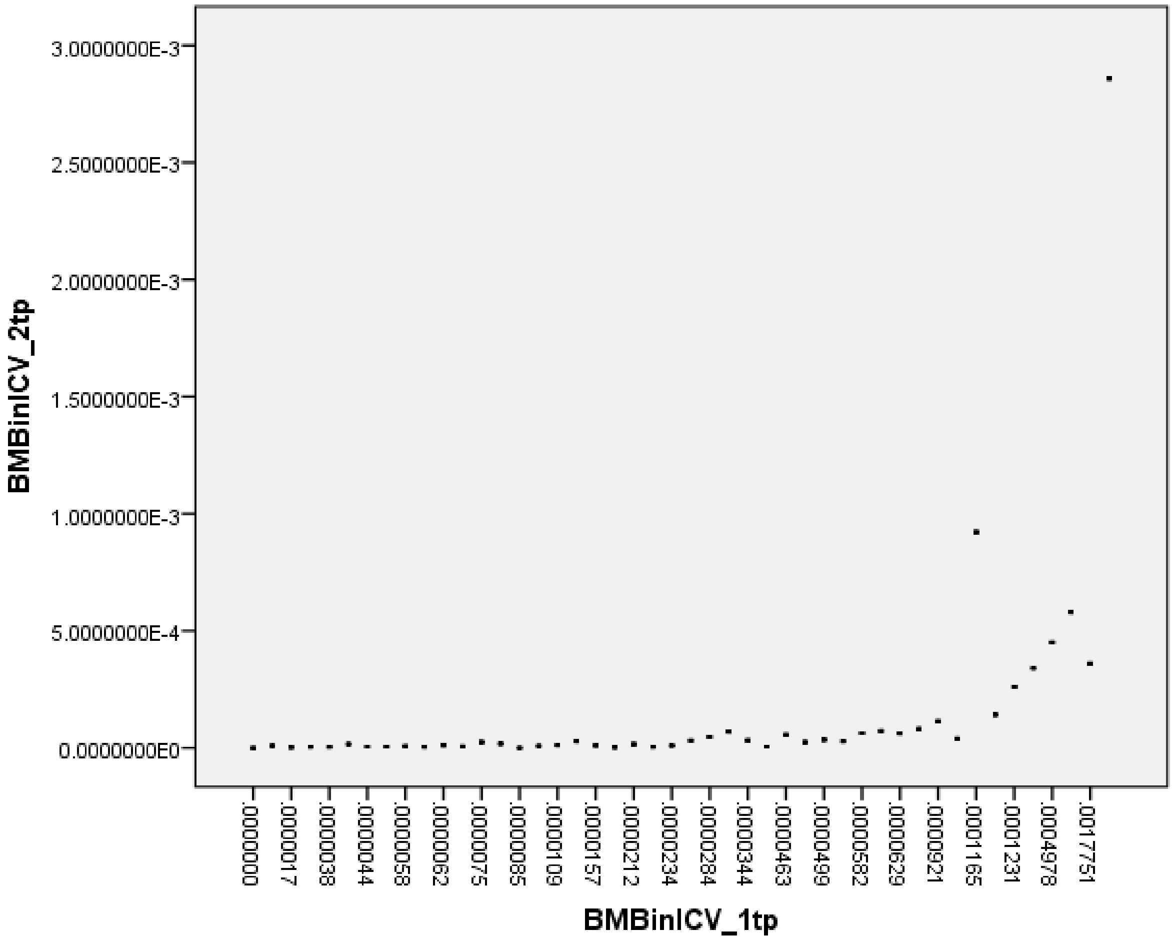

Figure S1.21. Volume of brain microbleeds and haemorrhages (adjusted by intracranial volume) at follow-up with respect to baseline

Volume of striatal iron deposition at 1-year follow-up (BGIDsinICV_2tp) examined with respect to the baseline measurements (BGIDsinICV_1tp)

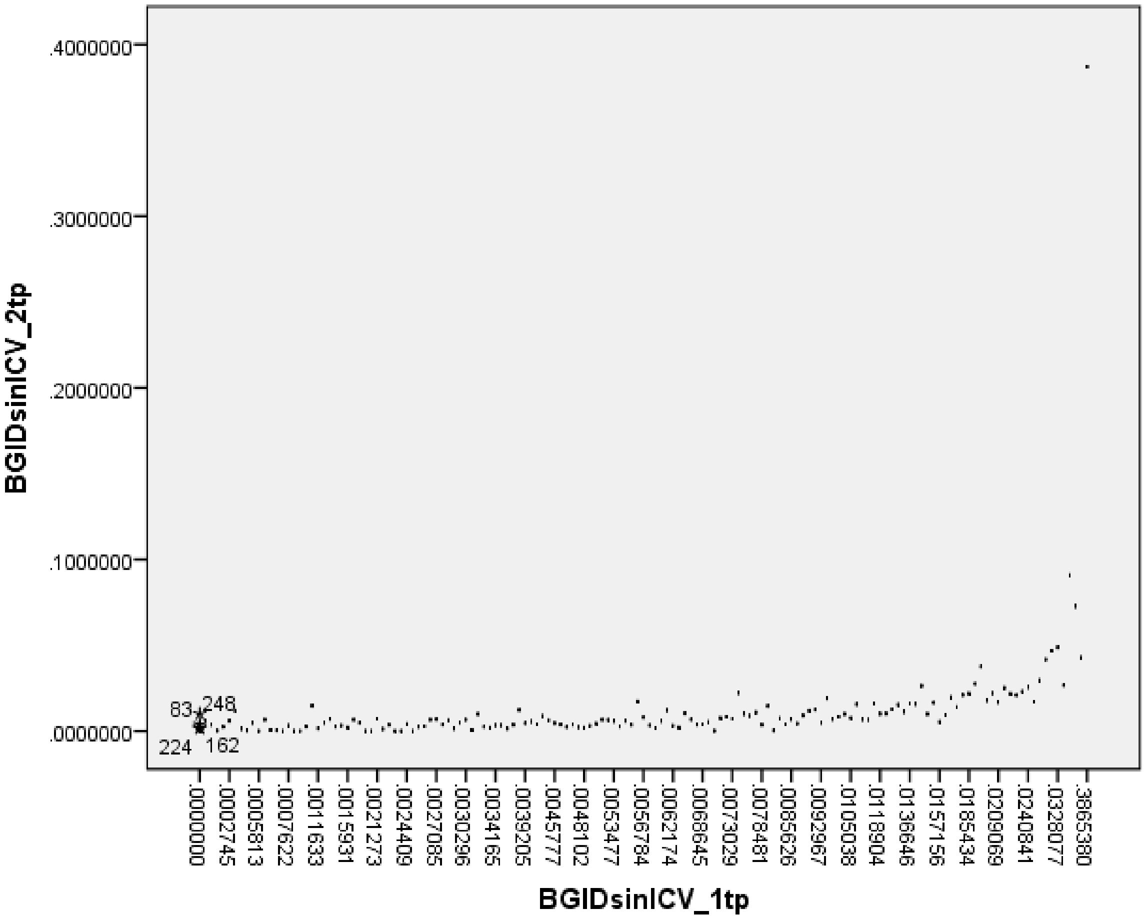

Figure S1.21. Volume of striatal iron deposition (adjusted by intracranial volume) at follow-up with respect to baseline

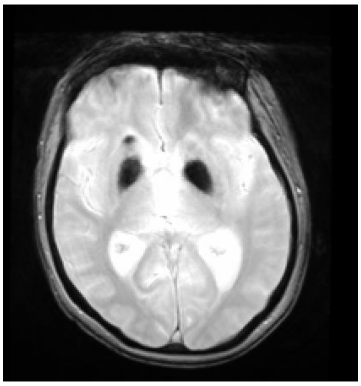

Figure S1.22. Axial gradient echo slice of the true outlier showing total mineralisation of the basal ganglia.

Volume of white matter hyperintensities at 1-year follow-up (WMHinICV_2tp) examined with respect to the baseline measurements (WMHinICV_1tp)

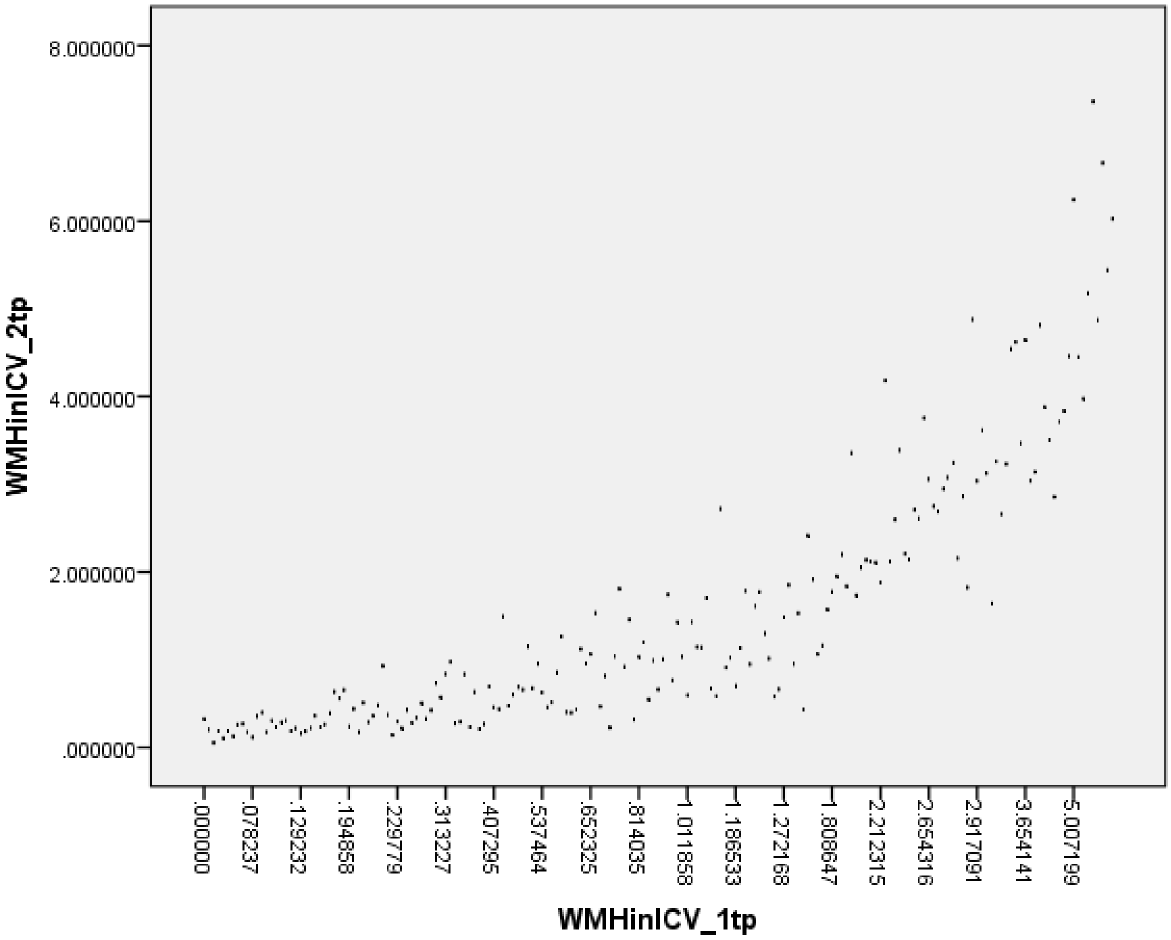

Figure S1.23. Volume of white matter hyperintensities (adjusted by intracranial volume) at follow-up with respect to baseline

Volume of total lesion (i.e. white matter hyperintensities and ischaemic stroke lesions old and recent) at 1-year follow-up (TLesioninICV_2tp) examined with respect to the baseline measurements (TLesioninICV_1tp)

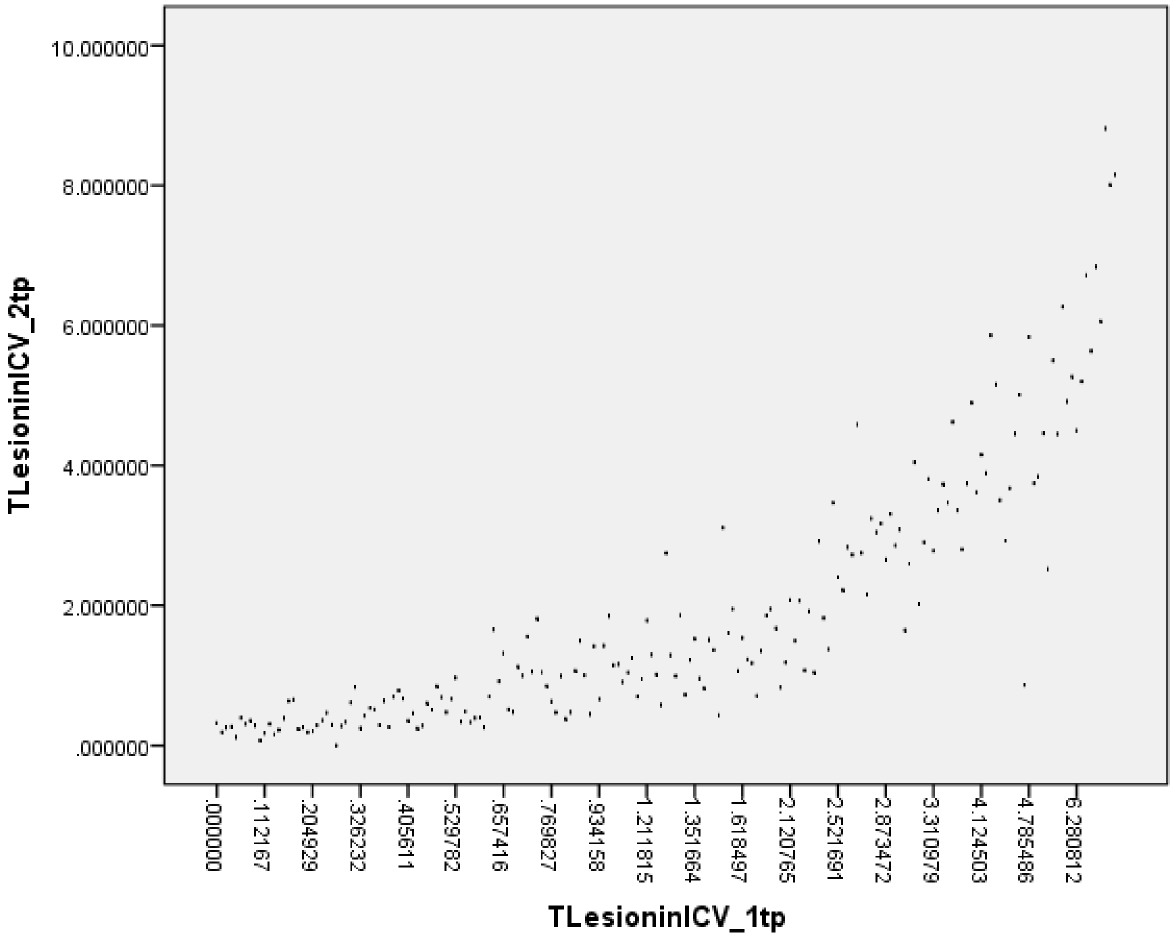

Figure S1.24. Volume of total lesion (i.e. white matter hyperintensities and ischaemic stroke lesions, old and recent) (adjusted by intracranial volume) at follow-up with respect to baseline

Analysis of Missing Values

The analysis of missing values was performed in IBM SPSS Statistics Ver 21 (Release 21.0.0.0). For all cognitive and imaging variables, we summarised and analysed the patterns of the missing values, and calculated means and standard deviation of missing data on each variable. In addition, to evaluate whether the missing values were associated to the vascular risk factors and lesion load at presentation, we calculated pairwise and regression statistics of missing values, assuming all data were missed at random, separately for the following cases: 1) hypertensive vs. normotensive, 2) with hyperlipidaemia and without, 3) smokers vs. no/ex-smokers, 4) who had a stroke of type lacunar vs. those who had a stroke of type cortical, 5) who had periventricular WMH extending into deep WMH and/or (early) confluent deep WMH (i.e. Fazekas periventricular WMH score ≥3 and/or Fazekas deep WMH score ≥2) at presentation vs. those who did not. The full results of these analyses are in the supplementary data excel spreadsheet SUPP_DATA_Missing_values_analyses_results.xlsx, available per request.

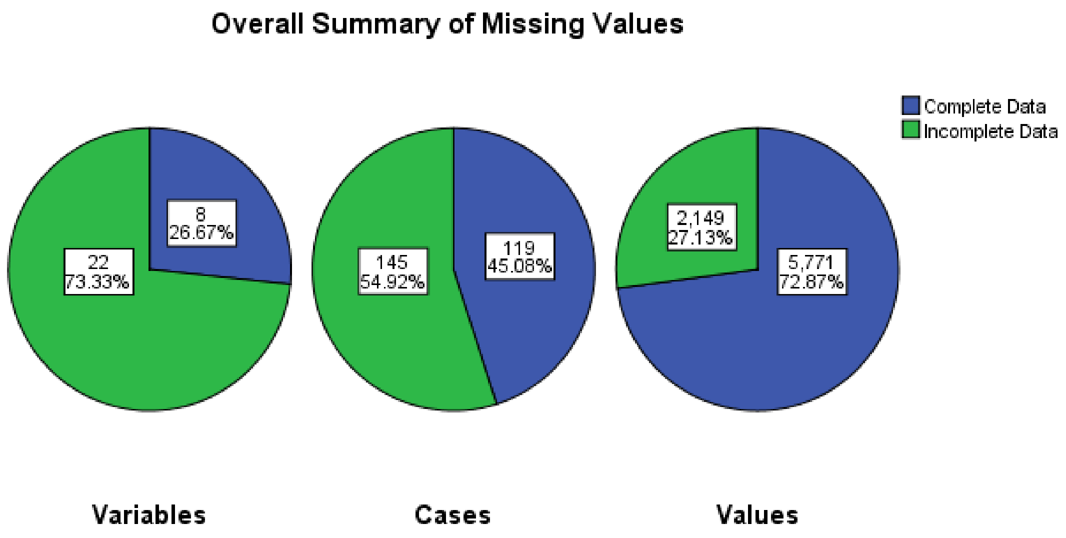

Figure S1.25. Summary of missing values

Variable Summary[a,b]

	Missing		Valid N	Mean	Std. Deviation
	N	Percent			
NART_2tp	124	47.0%	140	37.7286	10.42468
Visuospatial_2tp	113	42.8%	151	14.8742	1.45742
Language_2tp	113	42.8%	151	25.0265	1.50088
Fluency_2tp	113	42.8%	151	10.4371	2.66727
Memory_2tp	113	42.8%	151	21.0861	4.29254
Orientation_2tp	113	42.8%	151	17.4834	.97880

ACER_2tp	113	42.8%	151	88.9338	7.98387
NART_1tp	110	41.7%	154	35.1039	10.41126
Visuospatial_1tp	107	40.5%	157	14.8535	1.56817
Language_1tp	107	40.5%	157	24.6624	1.51723
Fluency_1tp	107	40.5%	157	10.3439	2.69547
Memory_1tp	107	40.5%	157	20.8217	4.40353
ACER_1tp	107	40.5%	157	88.0892	8.17232
Orientation_1tp	106	40.2%	158	17.3734	1.07954
TLesioninICV_2tp	75	28.4%	189	2.27123976	5.388711404
WMHinICV_2tp	75	28.4%	189	1.55289620	1.500178968
BGIDsinICV_2tp	75	28.4%	189	.010161297	.0300293188
BMBinICV_2tp	75	28.4%	189	.000036396	.0002271618
TLesioninBTV_2tp	74	28.0%	190	3.15499734	7.540125483
WMHinBTV_2tp	74	28.0%	190	2.16050173	2.110094163
BGIDsinBTV_2tp	74	28.0%	190	.014295826	.0425019854
BMBinBTV_2tp	74	28.0%	190	.000051688	.0003336687

a. Maximum number of variables shown: 25

b. Minimum percentage of missing values for variable to be included: 10.0%

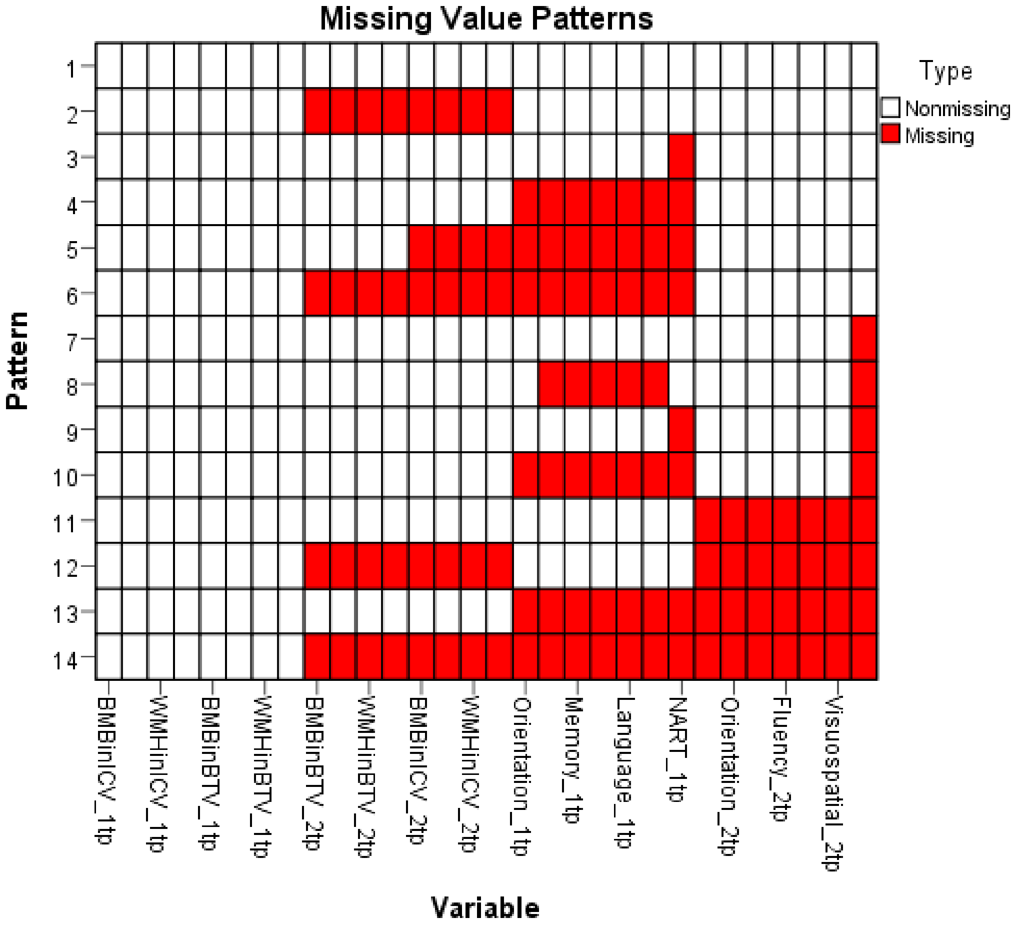

Figure S1.26. Missing value patterns

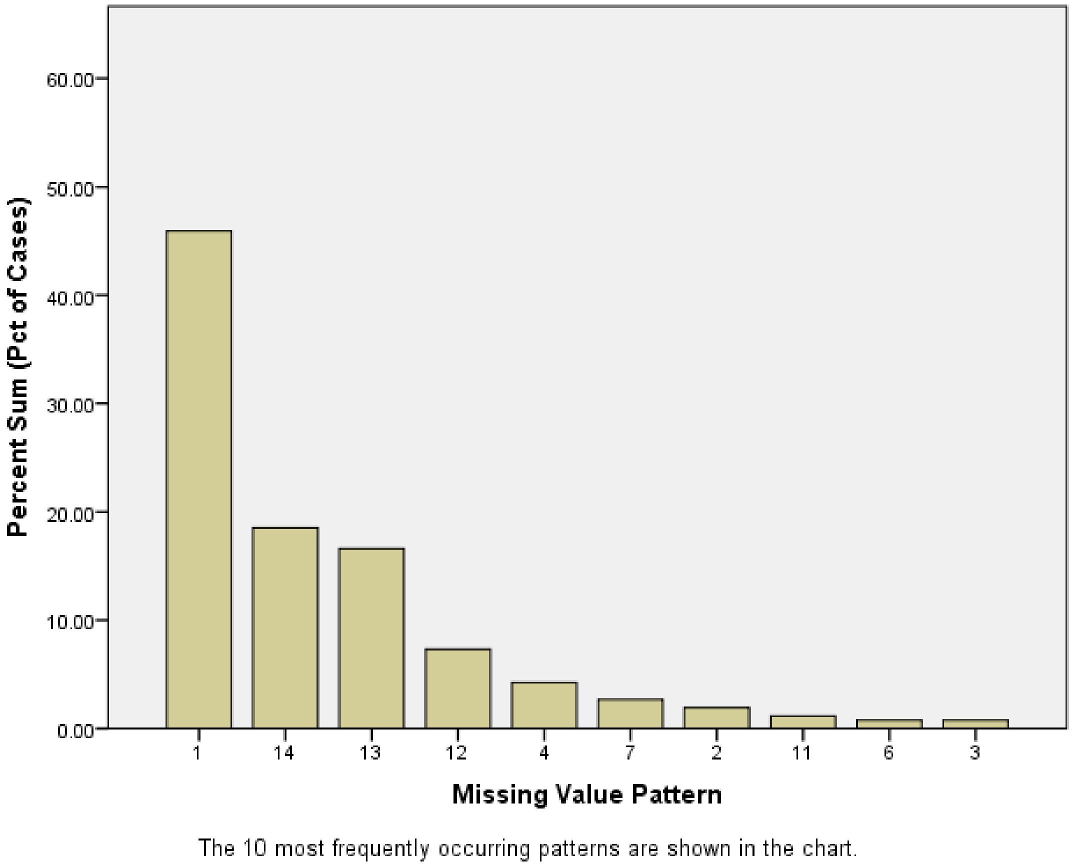

Figure S1.27. Percentage of cases missing for each of the 10 most frequent missing values

Belsley collinearity diagnostics

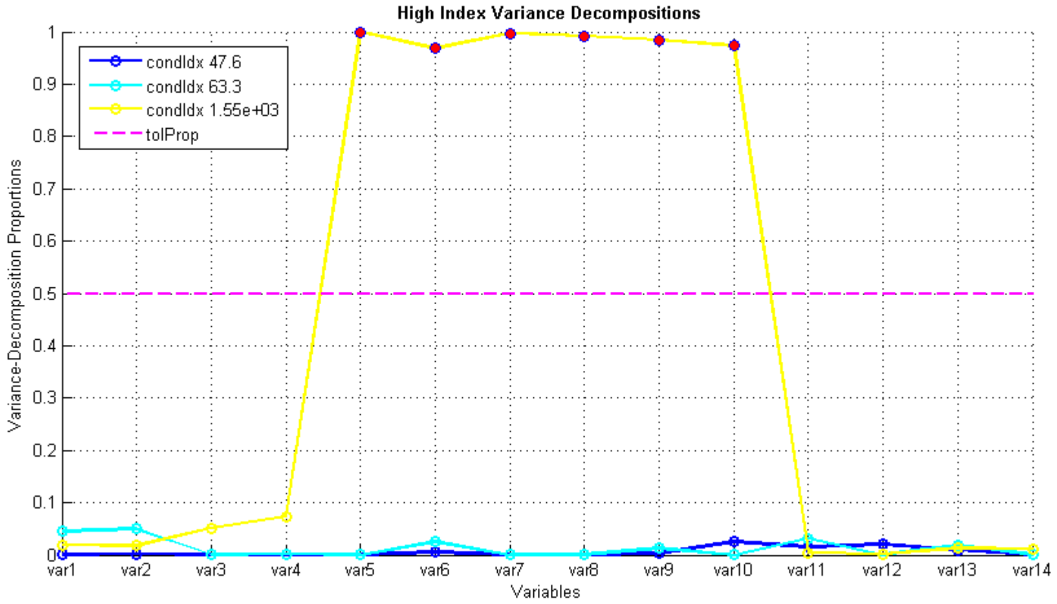

Figure S1.28. Variance decomposition results. As expected, cognitive variables are collinear amongst themselves but not with any other variable. Variables are: var1=T2swHypovol_1tp, var2=T2swHypovol_2tp, var3=BMB_and_Haem_1tp, var4=BMB_and_Haem_2tp, var5=ACER_1tp, var6=Orientation_1tp, var7=Memory_1tp, var8=Fluency_1tp, var9=Language_1tp, var10=Visuospatial_1tp, var11=NART_1tp, var12=Hypertension(Y/N), var13=Hyperlipidaemia(Y/N), var14=Smoker
